# Nutritional Value, Ethnopharmacology, Chemistry, and Biological Activities of Species of the Genus *Cnidoscolus*: An Updated Review

**DOI:** 10.3390/foods14122092

**Published:** 2025-06-13

**Authors:** Joice Barbosa do Nascimento, José Jonas Ferreira Viturino, Maria Alice Macêdo Ribeiro, José Galberto Martins da Costa

**Affiliations:** 1 Postgraduate Program in Biological Chemistry, Department of Biological Chemistry, Regional University of Cariri, Crato 63105-010, CE, Brazil; joicenascimento2010@live.com; 2 Natural Products Research Laboratory, Department of Biological Chemistry, Regional University of Cariri, Crato 63105-010, CE, Brazil; jjonasferreira5@gmail.com (J.J.F.V.); mariaalicemr716@gmail.com (M.A.M.R.); 3 Graduate Program in Biological Diversity and Natural Resources, Regional University of Cariri, Crato 63105-010, CE, Brazil

**Keywords:** biological potential, chemical composition, *Cnidoscolus* spp., ethnopharmacological applications, food consumption

## Abstract

Species belonging to the genus *Cnidoscolus* have been widely recognized for their diverse applications, including forage, oil production, latex, ornamental purposes, medicinal uses, and as nutritional sources. This study aimed to compile up-to-date information on the chemical, nutritional, and ethnopharmacological aspects, as well as the biological activities, of *Cnidoscolus* species, offering a critical overview of the current advancements in research on these plants. The reviewed literature indicates that *Cnidoscolus* species hold significant traditional use value, particularly in the treatment of conditions such as cancer, diabetes, and disorders affecting the uterus, prostate, ovaries, and kidneys, in addition to menstrual disturbances, inflammation, and general pain. Scientifically, their efficacy has been demonstrated in several contexts, including antinociceptive, antibacterial, anti-inflammatory, cytotoxic, antiproliferative, hypoglycemic, and antioxidant activities, among others. Additionally, certain species like *C. aconitifolius* have shown potential for human consumption, with leaves being eaten raw or cooked, while *C. quercifolius* demonstrates nutritional value in its seeds, which can be utilized in the development of functional foods. However, further studies are needed to focus on the isolation and characterization of bioactive compounds found in these species, as well as deeper investigations into the molecular and cellular mechanisms underlying their biological activities and assessments of the safety of long-term consumption in both humans and animals. Moreover, more extensive clinical and preclinical studies are essential to validate the proposed therapeutic effects and to support the safe and effective inclusion of these species in conventional treatment regimens.

## 1. Introduction

The family *Euphorbiaceae Juss.* represents one of the largest and most diverse lineages of angiosperms, comprising approximately 7600 species across about 300 genera. This family has a broad geographical distribution, predominantly found in tropical and subtropical regions of the Americas and Africa, occupying a wide range of habitats, from humid forests to drier ecosystems such as savannas and caatingas [1,2,3].

Among this rich diversity, the genus *Cnidoscolus* Pohl stands out, encompassing around 99 species mainly distributed in tropical and subtropical regions, especially in Mexico and Brazil, and exhibiting notable ecological adaptability [4]. *Cnidoscolus* species show a wide range of morphological and functional traits and are employed in various contexts, including urban afforestation, ecological restoration, timber and bioenergy production, forage, and traditional medicinal uses [5]. Ethnopharmacologically, several species are traditionally used in the treatment of a wide variety of ailments, such as disorders of the genitourinary and nervous systems, inflammatory conditions, pain, cancers (including uterine and prostate), diabetes, skin diseases, poisoning, dysentery, and eye problems [6,7,8].

The medicinal relevance of these plants is largely attributed to the presence of bioactive compounds with potential applications in pharmacological and biomedical sciences. Numerous studies have demonstrated that species of this genus exhibit anti-inflammatory, antinociceptive, antimicrobial, antioxidant, hypoglycemic, and hepatoprotective properties, reinforcing the therapeutic potential of *Cnidoscolus* [4,9,10,11]. In addition to their medicinal properties, some species, such as *Cnidoscolus aconitifolius* (Mill.) I.M. Johnst. and *Cnidoscolus chayamansa* McVaugh, commonly known as “chaya”, are notable for their significant nutritional value. The leaves of these plants are traditionally consumed in regions of Central America, especially in Mexico, and are rich in proteins, fiber, minerals, vitamins, and antioxidants, making them a promising alternative in combating food insecurity and malnutrition in tropical communities [12,13].

Despite the growing scientific and popular interest in the bioactive and nutritional properties of this genus, critical knowledge gaps remain in understanding the biochemical mechanisms underlying its therapeutic and nutritional effects. Given this context, the present study aimed to review and integrate the ethnopharmacological, nutritional, chemical, and biological aspects of species belonging to the *Cnidoscolus* genus. This is an integrative review in which we compile information, critically and comprehensively, on the state of the art in the chemistry, biology, and nutrition of the genus *Cnidoscolus*, bringing together results from experimental research and their implications. Furthermore, we identify gaps that deserve consideration and suggest perspectives for future studies, with a special focus on the sustainable use of these plants as resources for nutrition and public health.

The bibliographic research was conducted using the following databases: Science Direct, PubMed, Scielo, Scopus, Web of Science, and PMC. The search covered the literature published between 2000 and 2025, including articles in both English and Portuguese. Data collection occurred from January to March 2025, and searches were performed in both languages using the following keywords:

“*Cnidoscolus*” and (Ethnopharmacological applications or ethnomedicinal uses or biological activity or chemical composition or potential or nutritional value or food consumption). The preliminary analysis identified 2.509 publications, distributed among the databases as follows: 703 from Science Direct, 157 from PubMed, 246 from Web of Science, 12 from Scielo, 461 from Scopus, and 930 from PMC.

To ensure the relevance and quality of the sources included in this study, the following inclusion criteria were adopted: articles published between 2000 and 2025, written in English or Portuguese, and containing content related to the study topic. The exclusion criteria encompassed articles that did not align with the scope of the research, such as those that failed to report chemical, ethnopharmacological, nutritional, or biological properties of species within the genus *Cnidoscolus* spp. Studies considered to have low reliability, including drafts, preprints of submitted articles, duplicate publications, conference communications, theses, and dissertations, were also excluded. Following a rigorous screening process, 155 articles were selected for inclusion in the study, while 2.354 were excluded.

## 2. Genus *Cnidoscolus* spp.

**Figure 1:**
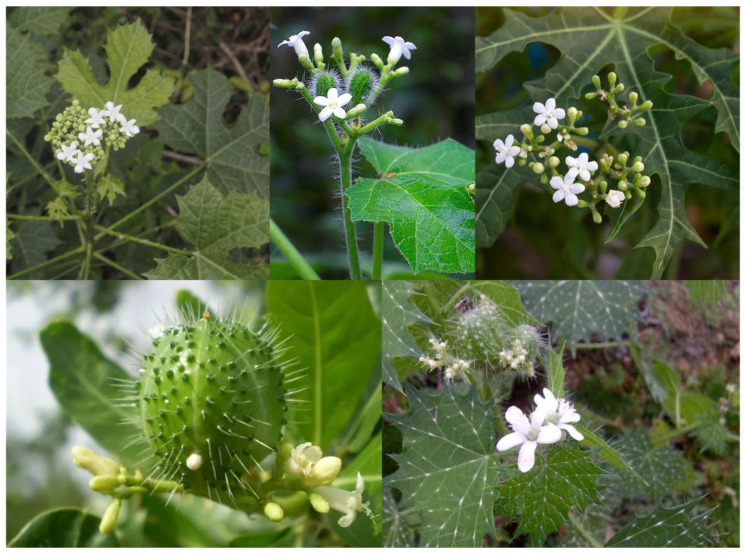
Flowering and fruiting in representative species of the genus *Cnidoscolus*.

The genus *Cnidoscolus* spp., belonging to the family *Euphorbiaceae* and subfamily *Crotonoideae*, comprises approximately 99 species, primarily distributed across Mexico and northeastern Brazil [14]. The northeastern region of Brazil is considered one of the centers of diversity for the genus, with 11 out of the 42 species recorded in the country [15]. The name *Cnidoscolus* derives from the Greek “κνίδη” (knide), meaning nettle, and “σκόλος” (skolos), meaning thorn or tip [16]. The genus was established by Johann Pohl in 1827 to include species characterized by a single whorl in the perianth and the presence of stinging trichomes across nearly all vegetative and floral parts (Figure 1) [17].

Despite their limited use and scarce economic exploitation, *Cnidoscolus* species have diverse applications. Some are used as forage plants, oilseed sources, latex producers, ornamentals, food sources, and in traditional medicine for the treatment of various diseases [5]. Additionally, the leaves of *C. aconitifolius* are consumed either raw or cooked [18], while the seeds of *Cnidoscolus quercifolius* are utilized as food for both humans and animals [19].

## 3. Ethnopharmacological Aspects

Among the species of the *Euphorbiaceae* family, the genus *Cnidoscolus* stands out in ethnopharmacological studies, being associated with multiple uses in traditional medicine, as shown in Table 1. According to the literature, the genus holds significant cultural relevance and is widely used across various medicinal traditions, particularly in Asia, the Americas, and Africa. The leaves of *Cnidoscolus aconitifolius* are the most commonly used parts of the plant, with therapeutic applications extensively documented in both Eastern and American countries. In Mexico and Africa, especially among traditional communities, the leaves are used in the treatment of diabetes [20,21].

In Nigeria, the sap or stem of *C. aconitifolius* is used to relieve symptoms related to headaches and eye problems [22], and it is traditionally associated with the treatment of sickle cell anemia [23]. In India, infusions made from its leaves are commonly used to treat diabetes, eye diseases, skin ailments, and metabolic disorders [8]. In Mexico, *C. aconitifolius* is also utilized in the treatment of gynecological issues and to promote the production and flow of breast milk [24]. In Colombia, a decoction of the whole plant is used as an antivenom [25].

In Brazil, *C. urens* and *C. quercifolius* have significant traditional uses. Preparations made through the decoction and maceration of their roots and leaves are employed to treat disorders of the genitourinary and nervous systems, musculoskeletal tissue injuries, poisoning, infectious and parasitic diseases, as well as skin and subcutaneous tissue conditions [26]. According to Magalhães et al. [27], *C. quercifolius* is effective in relieving toothaches, while *C. urens* is traditionally used to treat constipation, toothaches, decreased sexual desire, infectious diseases, and dermatophytosis. Moreover, decoctions made from the stem bark of *C. phyllacanthus* (synonym of *C. quercifolius*) are traditionally recommended as anti-inflammatory agents for the treatment of ovarian and prostate problems [28].

In the Central-West region of Brazil, *C. urens* is used to treat skin conditions (such as superficial mycoses, itching, infections, and wounds), uterine infections, injuries, and stomach disorders [29]. In the north of Brazil, its roots are used to treat kidney stones and albuminuria [30]. In traditional communities of the northeast, the roots are commonly employed to treat cancer (uterine and prostate), dysentery, gynecological disorders, hemorrhages, inflammations, and general pains, as well as kidney and menstrual disorders [31]. In a quilombola community in Bahia, *C. urens* is used to relieve dental inflammations [32].

Although few studies are available, other *Cnidoscolus* species have also been cited in ethnobotanical research. *C. obtusifolius*, for instance, is traditionally used in the treatment of various conditions, including cancer, tumors, and hepatic and uterine inflammations [26]. *C. tubulosus* is employed in some communities to induce vomiting [33]. *C. chayamansa* has been studied for its potential cardiovascular protective effects [34] and is also used in the treatment of diabetes and urinary tract infections [7].

Furthermore, traditional knowledge indicates that the roots of *C. infestus* are highly valued for treating urinary tract disorders and ovarian inflammations [28,31]. *C. multilobus*, in turn, is used as a mouthwash for the treatment of gingival diseases [35].

Although species of the genus are widely used in traditional medicine across different continents and hold strong cultural relevance, clinical evidence supporting these ethnopharmacological claims remains limited. Most available data are based on anecdotal reports or ethnobotanical surveys, with minimal pharmacological validation under controlled conditions. Therefore, there is an urgent need for rigorous clinical trials to assess their safety, efficacy, and mechanisms of action in humans. Advancing this line of research will be essential not only to substantiate traditional uses but also to support the integration of these plants into modern healthcare systems.

**Table 1:** Traditional medicinal uses of species from the genus *Cnidoscolus*.

Species Name	Plant Part	Usage Form	Biological Activity in Traditional Medicine	Reference
*Cnidoscolus aconitifolius* (Mill.) I.M. Johnst. (Chaya)	Whole plant	-	Diabetes	[20]
	Leaves	Infusion: oral	Diabetes	[21]
	Leaves and stem	-	Irritated eyes and headaches	[22]
	Leaves	-	Control sickle cell anemia	[23]
	Aerial part and leaves	Infusion: oral	Diabetes, eye problems, skin diseases, improved digestion	[8]
	Leaves	-	Promotes the production and flow of breast milk	[24]
	Whole plant and leaves	Decoction: oral	Antivenom activities	[25]
*Cnidoscolus quercifolius* Pohl (Favela) and *Cnidoscolus urens* (L.) Arthur (Cansansão)	-	-	Disorders of the genitourinary system, nervous system, and musculoskeletal tissue; injuries; poisoning; infectious and parasitic diseases; diseases of the skin and subcutaneous tissue	[26]
*C quercifolius*	Bark (stalk)	Maceration in water	Pain, toothache	[27]
*C. urens*	Root and whole plant	Juice from the bark, latex	Constipation, toothache, reduced sexual urge, infectious disease, dermatophytosis	[27]
*Cnidoscolus phyllacanthus* (Mull.Arg.) Pax & K.Hoffm (synonym of *C. quercifolius*) (Favela)	Stem bark	Decoction: oral	Anti-inflammatory effects in the ovaries and prostates	[28]
*C. urens*	Root	Decoction, maceration	Furuncle, “impingem” (superficial mycoses of the skin), blood purifier, stomach pain, itching, skin infection, uterine infection, skin wounds, lesion	[29]
	Root	Infusion: oral	Albuminuria, kidney stones	[30]
	Root	-	Cancer, uterus, prostate, dysentery, ovaries, hemorrhage, inflammation and pains in general, menstruation, kidneys	[6]
	-	-	Dental inflammation	[32]
*Cnidoscolus obtusifolius* Pohl (Faveleira)	Leaf	Ethanoic extract	Cancer, tumor, liver, uterus inflammation	[26]
*Cnidoscolus tubulosus* (Mull. Arg.) I.M. Johnst	Seed	Maceration: oral	Vomit	[33]
*Cnidoscolus chayamansa* Mc Vaugh (Chaya)	-	-	Cardioprotection	[34]
	Leaves	Infusion: oral	Diabetes, urinary tract infection	[7]
*Cnidoscolus infestus* Pax & K. Hoffm (Urtiga-de-boi)	Root	-	Urinary afflictions, ovarian inflammation	[31]
	Root	Decoction: oral	Urinary and ovarian inflammations	[28]
*Cnidoscolus multilobus* (Pax.) I.M. Johnston (Ortiga)	Leaves	Mouthwash	Gum diseases	[35]

## 4. Nutritional Considerations and Functional Potential of *Cnidoscolus* spp.

Species of the *Cnidoscolus* genus, particularly *C. aconitifolius* and *C. chayamansa*, have been widely used as food in various communities, notably in Nigeria, Central America, and the Caribbean [36]. The leaves of these species are rich in proteins, fibers, lipids, vitamins, carbohydrates, and minerals, making them a source of essential nutrients with potential applications in human and animal nutrition [37,38,39]. Additionally, they contain important bioactive compounds such as flavonoids, tannins, alkaloids, and phenolic acids, which confer therapeutic properties, including antioxidant, hepatoprotective, immunomodulatory, neuroprotective, hypolipidemic, and antianemic activities [40,41,42,43,44,45,46,47]. Thus, incorporating these species into the diet can provide not only nutritional benefits but also aid in the prevention of various diseases.

Studies indicate that *C. aconitifolius* contains significant levels of water (79.80 g/100 g), energy (64 kcal/100 g), protein (6–23.96%), lipids (1.30 g/100 g), fiber (9.89–37%), carbohydrates (approximately 65%), ash (2.60–9.27%), and minerals such as iron (130.87 mg/kg), calcium (55 mg/g), magnesium (89 mg/g), potassium (22 mg/g), and phosphorus. The presence of both water-soluble (such as folic acid, thiamine, riboflavin, and niacin) and fat-soluble vitamins (A, E, and K), especially the high concentration of vitamin A (143.2 mg/100 g), stands out as a major nutritional highlight [12,48]. These findings are corroborated by Oyagbemi et al. [49], Kayode [50], Adamu et al. [51], and Gobena et al. [52], who also identified high levels of these nutrients, suggesting that this species is a promising resource in combating malnutrition, particularly in arid regions.

According to Wong et al. [48], only 100 g of *C. aconitifolius* leaves is sufficient to meet the daily energy, protein, and lipid requirements, with only 12 kcal/day. This supports the theory proposed by Schwarcz et al. [53] that *C. aconitifolius* leaves significantly contributed to the protein intake of the Maya people during periods and in regions where animal protein sources were limited.

Among the studied forms, John and Opeyemi [54] demonstrated that the extract from *C. aconitifolius* leaves had the highest protein content (3.76%), while the residue after extraction contained 2.57%. Fresh leaves contained 0.47% fat, which decreased in processed extracts and residues. Leaf residues after extraction showed the highest energy value (89.79%), carbohydrate content (22.96 ± 0.03%), and fiber content (4.07 ± 0.05%). Additionally, the mineral composition varied, with calcium, iron, magnesium, and phosphorus levels decreasing after processing. Setiasih et al. [39] also found that leaves without stems had a higher quality, with the crude protein content reaching 29.39% compared to 23.79% in leaves with stems.

The plant’s functional potential has also been demonstrated in food formulations. Partial substitution of wheat flour with *C. aconitifolius* and amaranth flour in cookie production significantly increased protein (9.21–10.10%) and fiber (5.34–6.63%) contents without compromising sensory acceptability. Furthermore, baking reduced the antinutritional content by decreasing oxalates, indicating the feasibility of using the plant in developing functional foods [55].

In terms of food safety, antinutrient levels such as those of phytic acid and oxalates remain within acceptable limits, and toxic compounds have not been detected [56]. Additionally, John and Opeyemi [54] verified that effective processing of *C. aconitifolius* leaves can minimize antinutritional components. Thermal processing reduced oxalate levels from 78.33 mg/100 g in fresh leaves to 31.67 mg/100 g in the extraction residue. Similarly, phytates decreased from 50.00 mg/100 g to 8.00 mg/100 g, improving the undesirable properties associated with their consumption.

A reduction in antinutritional content was also observed by Adamu et al. [51] when analyzing fresh, blanched, and cooked leaves. According to their results, cooking reduced antinutrients: oxalates dropped from 62.71 to 30.04 mg/100 g, phytates from 77.17 to 28.64 mg/100 g, and hydrogen cyanide from 171.22 to 0.00 mg/100 g. The saponin content slightly increased from 218.50 to 220.30 mg/100 g after cooking.

From a zootechnical perspective, dried *C. aconitifolius* leaf meal has good nutritional value and digestibility, making it suitable for use in pellets for ruminant supplementation [57]. High levels of total digestible nutrients and digestible energy were observed, indicating its viability as an energy source. Furthermore, the species contributes to more sustainable ruminant production practices by reducing methane emissions [58] and enhancing ruminal fermentation [59], although its rapid nitrogen release may limit its combination with other energy sources such as cassava [60].

Another species with nutritional potential is *C. chayamansa*. Jiyil et al. [61] reported that its leaves offer good hydration and energy potential due to their moisture content (12.62 ± 0.00), high carbohydrate levels (27.48 ± 0.02), crude fiber (25.18 ± 0.02), protein (18.63 ± 0.01), ash (11.68 ± 0.01), and low lipid concentration (4.40 ± 0.01). Mineral analysis also showed richness in essential minerals such as zinc (102.5 ± 0.5 mg/kg), sodium (0.018 ± 0.001 mg/kg), magnesium (0.866 ± 0.001 mg/kg), potassium (2.41 ± 0.05 mg/kg), calcium (6.67 ± 0.05 mg/kg), phosphorus (0.37 ± 0.01 mg/kg), and iron (0.063 ± 0.01 mg/kg), consistent with results from Olaposi and Adunni [62].

It was also demonstrated that the protein from *C. chayamansa* leaves had a purity of 63.52%, high digestibility, and a complete essential amino acid profile, meeting the standards of the Food and Agriculture Organization (FAO). The protein efficiency ratio was 2.87, confirming its quality. These attributes highlight its potential as a nutritious and protein-rich food source for both human and animal health [63].

Wongnhor et al. [64] investigated the effects of including *C. chayamansa* leaf flour in the diet of native Thai chickens (Pradu Hangdum). According to the results, there was no significant impact on total feed intake, but there was a decrease in body weight gain with increasing amounts of flour (10%, 20%, and 30%). This effect suggests that despite the species being nutrient-rich, its inclusion at higher levels may have limiting impacts on growth performance. A linear increase in the counts of heterophils, eosinophils, and monocytes was also observed in the groups fed with this species, indicating a potentially increased immune response. Although beneficial for overall health, the amount used must be carefully determined to avoid negatively affecting growth performance.

Poot-López et al. [65] investigated the use of *C. chayamansa* leaves as a dietary supplement for tilapia and found that *C. chayamansa* alone did not promote significant weight gain. However, when supplemented as part of a balanced diet, it increased weight gain from 190.9 g to 280.9 g, demonstrating a 47.1% increase. These results suggest that *C. chayamansa* alone does not provide sufficient nutrients for optimal tilapia growth but can act as a complementary food, reducing feeding costs and providing economic returns in aquaculture systems.

Moreover, *C. chayamansa* was shown to offer beneficial effects on glucose metabolism and kidney health in Wistar rats, without negatively affecting nutritional biomarkers such as serum proteins, alkaline phosphatase, alanine aminotransferase, aspartate aminotransferase, total cholesterol, or triglycerides [66].

The seed oil of *C. quercifolius* also demonstrates good nutritional quality, with low acidity (0.78%), a low peroxide value (1.13 mEq/1000 g), and 53.56% linoleic acid [67]. A study by Medeiros et al. [68] investigated the nutritional implications of using this vegetable oil in the diet of dairy goats and its effects on cheese production. The oil, with up to 70% lipid content and rich in linoleic acid (41.6%), was included at 4% in the diet of dairy goats, resulting in cheeses with higher levels of unsaturated fatty acids (C18:2 and C18:1). Besides the nutritional benefits, the oil also improved sensory aspects such as the hardness and appearance of the cheese without altering its overall quality, proving to be a promising supplement in goat production.

Similarly, Medeiros et al. [69] found that adding the oil to goat diets increased the desirable fatty acid content in cheese from 42.43% to 50.86%, improving its nutritional profile without increasing the total fat content.

Oliveira et al. [70] highlighted the potential of *C. phyllacanthus* as a nutritious forage for ruminants in tropical regions, with benefits for digestion and animal productivity. The authors observed that during the vegetative stage, *C. phyllacanthus* presented 143.2 g/kg of crude protein, high gas production (206.02 mL/g), good ruminal degradability (61.63%), and digestibility of 627.1 g/kg, in addition to providing essential minerals.

*C. quercifolius* hay was included in mineral salt licks for lambs, and although it did not significantly affect feed intake or weight gain, supplemented animals achieved daily gains adequate for slaughter, suggesting its viability during dry seasons [71]. Oliveira et al. [72] also demonstrated that the leaves of *C. quercifolius* processed into forage salt, especially with 1% to 3% NaCl, increased the intake and digestibility of crude protein, ether extract, and non-fibrous carbohydrates in sheep. Dry matter digestibility reached 63.66% with a hay-based diet. However, salt levels above 3% reduced consumption.

Besides its nutritional value for animals, the species also shows significant functional potential. The potential of *C. quercifolius* seed cake flour for human consumption was verified by Ribeiro et al. [73]. The results demonstrated that the flour is rich in proteins, lipids, fibers, and polyphenols. Cookies made with 50% substitution of refined flour with seed flour showed an increase in nutrient content and linoleic acid (97.50 g/kg) without compromising sensory quality, thus being a promising source for the production of functional products.

Another species of the genus that can be a valuable addition to the diet is *C. aurifolia*. This species contains a high protein content (59.45 ± 0.07%), along with significant amounts of crude fiber (11.80 ± 0.08%) and fat (7.60 ± 0.11%), and has a low carbohydrate content (2.27 ± 0.01%). It also showed the presence of several essential minerals, including calcium (96.30 mg/100 g), iron (34.10 mg/100 g), magnesium (63.50 mg/100 g), potassium (98.34 mg/100 g), and zinc (17.40 mg/100 g). The antinutrient analysis indicated low levels of harmful compounds, such as hydrogen cyanide (0.32 mg/100 g) and oxalate (404.80 ± 0.11 mg/100 g), suggesting that the consumption of these leaves is not nutritionally harmful and does not present adverse health effects [74]. However, other studies on its nutritional potential were not found.

Despite the vast nutritional potential and applicability of *Cnidoscolus* species in human and animal diets, further studies are needed to validate their functional efficacy. Clinical studies in humans are still scarce, and long-term consumption effects remain to be fully elucidated. Moreover, to ensure their nutritional efficacy, standardization of preparation and consumption methods is required, as boiling, drying, and other techniques can affect nutrient availability.

## 5. Chemical Aspects

The species of the genus *Cnidoscolus* present a great diversity of bioactive chemical compounds from their secondary metabolism with applicability in the most diverse sectors of the economy, mainly in the food sector as a source of primary nutrients such as proteins, carbohydrates, and minerals, and in health, being used in the production of medicines and in the treatment and cure of various diseases among traditional peoples and communities. Among the main species most studied regarding their chemical composition and bioactivities, the species of *C. aconitifolius, C. chayamansa*, and *C. quercifolius* stand out, which are described in more detail throughout the manuscript.

Among the main parts of plants of the genus used as a source of raw material for the development of studies are the leaves, stems, and roots, as well as fruits and seeds. The main extraction methods used to evaluate the bioactivity of the compounds present in the plant material of the *Cnidoscolus* spp. were maceration in which the extracting agent (solvent) remains in contact with the plant material for a period, Soxhlet, chemical fractionation by a polarity gradient, and hydrodistillation for extraction of essential oils, among other techniques used.

For the analysis of the chemical composition of the species belonging to the genus *Cnidoscolus* spp., the main qualitative and quantitative methods for analysis of the chemical composition of the extracts and other samples from the species of the genus *Cnidoscolus* found in this study were chemical prospecting to identify the main classes of metabolites present in the samples, this being common in most studies, followed by the quantification of phenols and flavonoids and other classes such as alkaloids and saponins, and finally the use of more advanced chromatographic techniques such as HPLC, GC/MS, GC/FID, UHPLC, NMR, and thin-layer chromatography (TLC), among others, that allowed the identification and quantification of compounds present in the plant samples, as well as chromatographic methods for the chemical isolation of active substances. In this work, the authors decided to present this information by plant species, separately, as can be seen in the sequence.

In general, the use of chromatographic techniques, especially hyphenated ones, combined with spectroscopic methods such as uni- and bidimensional NMR, enables the characterization and identification of both secondary and primary metabolites in plants of medicinal and nutritional interest. On the other hand, the experimental activities concerning the use of these analytical techniques may present limitations that directly affect the reliability and reproducibility of the results obtained. For example: (1) solvent choice, (2) analysis time, and (3) extraction temperature can influence the chemical composition of the extract, potentially favoring certain metabolite classes over others. Another point to consider is the disproportionate emphasis placed on identifying certain substances, often overlooking substances present in low concentrations, which may actually be what are sought.

These methodological directions directly impact the verification of biological activities, such as antioxidant, antimicrobial, or anti-inflammatory properties. It is a fact that poorly executed extraction may lead to an underestimation of the therapeutic potential of a plant, especially when biological assays are conducted with crude or poorly characterized extracts. On the other hand, the correlation between substances identified by techniques such as HPLC/UPLC or GC-MS and the biological impacts observed often requires further validation. The NMR technique, widely used, despite being robust in structural elucidation, still needs to be more effective for quantitative analysis or for complex matrices, such as plant extracts, so as not to limit its applicability in integrated studies of the clear relationships between chemical structure and biological function.

Regarding information in the area of functional foods or nutraceuticals, the limitations are even more pronounced. Key elements, such as variability in the chemical composition of plants caused by factors such as seasonality, geographical origin, and processing methods, are not captured by point-of-use analytical methods. This observation compromises the standardization and effectiveness of products, negatively impacting their safety and efficacy. Therefore, more comprehensive and standardized analytical protocols are required to account for the chemical complexity of plants along with their biological activities in research on food and diverse products based on medicinal plants.

### 5.1. Quantification of Secondary Metabolites

Several studies have conducted quantitative analyses of the main classes of secondary metabolites present in leaf extracts of *Cnidoscolus aconitifolius*. Among the compounds identified, tannins stand out, with concentrations ranging from 18.00 mg/kg to 38.70 mg GAE/g [37,50,75], as well as condensed tannins at 2.40 mg GAE/100 g [76]. Alkaloids were reported in amounts ranging from 13.83 mg/kg to 0.316 g/g [37,50,75,77], and saponins in concentrations from 4.45 mg/g to 0.234 g/g of sample [37,75]. The leaves also contained cyanogenic glycosides at concentrations around 5.02 mg/g [75].

Flavonoid contents showed a wide range, from 12.53 mg/kg to 69.3 µg QE/mg, with the highest levels observed in fresh leaves and concentrated extracts [50,77,78,79,80]. Total phenols also exhibited a broad variation, from 3.30 mg/g to 405.56 mg GAE/g [10,37,75,77,80]. In fractionated extracts, the phenol content ranged from 21.46 µg GAE/g to 28.18 mg GAE/100 g [10,81], while in leaf-based beverages, values reached up to 1359 mg GAE/L [44].

Furthermore, the chemical profile of the extracts varied significantly depending on the extraction method and the type of sample (fresh or cooked leaves). Rodrigues et al. [42], analyzing the influence of extraction methods on the total phenol content in *C. aconitifolius* leaf extracts, reported that the Soxhlet method yielded the highest total phenol content, with 82 mg GAE/g of extract. Babalola [82], in a comparative analysis of fresh and cooked leaves and extracts of *C. aconitifolius*, found higher concentrations of alkaloids (108.33 mg/100 g), carotenoids (1906.6 mg/100 g), flavonoids (260.00 mg/100 g), phenols (25.50 mg GAE/100 g), and saponins (225.00 mg/100 g) in fresh leaves compared to other samples. The stem bark, in turn, showed higher concentrations of phenols (285.1 mg GAE/100 g) and flavonoids (51.1 mg GAE/100 g) when cooked [18]. Kuti [83] evaluated the phenol and flavonoid contents in raw and cooked *C. aconitifolius* leaves, reporting phenol levels of 2906.2 and 2241.4 µg/g of sample and flavonoid levels of 75.1 and 62.6 µg/g of sample, respectively.

Other species of the genus have also been analyzed. In *C. chayamansa*, the phenol content ranged from 0.42 µg GAE/g to 71.3 mg GAE/g and the flavonoid content ranged from 4.45 µg QE/g to 42.7 mg CE/g [83,84,85,86]. In oil extracted from *C. quercifolius* seeds, the phenol content ranged from 108.11 to 324.92 mg GAE/100 g, flavonoids were found at 18.70 mg RE/g, tocopherols at 21.3 mg/100 g, and *β*-sitosterol at 131.6 mg/100 g [19,67,87].

For *C. urens*, extracts presented phenol contents ranging from 20.69 mg GAE/g (stem) to 62.04 mg GAE/g (root), while flavonoids ranged from 6.20 mg QE/g (stem) to 18.81 mg QE/g (root) and 20.49 mg QE/g (leaf) [88]. Finally, in *C. multilobus*, a high concentration of phenols was reported, with 549.91 mg GAE/g in the leaf extract [89]. *C. phyllacanthus* showed phenols at approximately 135.8 mg GAE/g in crude leaf extracts, tannins at 72 ppm, alkaloids accounting for 0.62% of the extract, and anthocyanins at 0.12 mg/100 g [90].

These findings highlight the diversity and richness of bioactive metabolites in species of the genus *Cnidoscolus*, with significant variations among plant parts, preparation methods, and analyzed species. This underscores the potential of these plants for food and pharmaceutical applications.

### 5.2. Chemical Compounds Identified

Species of the genus *Cnidoscolus*, particularly *C. aconitifolius*, *C. chayamansa*, and *C. quercifolius*, have been extensively investigated for their phytochemical profiles [9,12,37,42,47,91,92,93,94,95]. Most of these studies have focused primarily on leaf extracts, which have revealed a wide range of compounds belonging to various classes of secondary metabolites. These compounds are detailed in Table S1 (Supplementary Materials).

Chromatographic analysis of *C. aconitifolius* leaves has revealed a variety of compounds across several classes, including flavonoids (e.g., quercetin and kaempferol), phenolic acids (e.g., ferulic, gallic, caffeic, and chlorogenic acids), coumarins (e.g., scopoletin and umbelliferone), lignans, xanthones, stilbenes, triterpenes (e.g., lupeol and *β*-amyrin), sterols (e.g., stigmasterol and *β*-sitosterol), fatty acids (e.g., palmitic and linoleic acids), carotenoids (e.g., *β*-carotene), and vitamins (e.g., vitamin E and riboflavin), among others [10,42,44,76,81,83,96,97,98,99,100,101,102,103].

In *C. chayamansa*, compounds identified include flavonoids, phenolic acids, triterpenes, sterols, indole alkaloids, vitamins, and other bioactive metabolites [81,83,84,104,105]. Similarly, studies on *C. quercifolius* have reported the presence of compounds from various classes, including phenols, flavonoids, tannins, xanthones, lignans, coumarins, anthraquinones, terpenes, and sterols, as well as saturated and unsaturated fatty acids; mono-, di-, and triglycerides; sesquiterpenes; and diterpenes, in extracts from leaves, stems, roots, flowers, and seed oil [5,19,67,87,106,107,108,109,110,111,112,113,114,115,116].

In *C. tehuacanensis*, the main compounds identified are triterpenes such as *β*-amyrin, lupeol, and their acetylated derivatives, in addition to sterols like *β*-sitosterol and long-chain fatty alcohols, exemplified by 1-octacosanol [117]. The analysis of *C. texanus* revealed 26 compounds, including 15 flavonoids, 3 coumarins, 3 p-coumaric acid derivatives, 4 triterpenoids, and 1 phytosterol [118]. In *C. urens*, the seed oil was found to be rich in saturated and unsaturated fatty acids, including palmitic, stearic, oleic, and linoleic acids. In leaf and bark extracts, additional compounds such as other fatty acids, phytols, ethyl esters of fatty acids, and squalene were also identified, indicating a complex lipid composition [119,120]. Finally, in *C. phyllacanthus*, the seed oil exhibited a similar profile, composed primarily of fatty acids such as palmitic, linoleic, oleic, and stearic acids [121].

The presence of compounds belonging to the reported classes in species of the genus *Cnidoscolus* highlights their potential applications across various economic sectors, especially in the food industry as a source of nutrients and minerals. Additionally, their antioxidant effects, primarily attributed to phenolic and flavonoid compounds, further underscore their value, along with their pharmacological applications. These include antimicrobial activity, particularly associated with tannins, terpenes, and saponins, as well as anti-inflammatory effects linked to phenols, polyphenols, and fatty acids.

### 5.3. Isolated Chemical Compounds

From the extracts of the leaves of *C. chayamansa*, it was possible to isolate the fatty acid compounds 8-methyl-6-nonanoic acid, characterized as a methyl ester; moretenol; moretenyl acetate; moretenone; *β*-amyrin acetate; ergost-5-en-ol; stigmasterol; *β*-sitosterol; kaempferol-3;7-dimethyl ether; 5-hydroxy-7;3′;4′-trimethoxyflavanone; quercetin; kaempferol stigmastadiene; *β*-amyrin acetate; amyrenone; lupeol acetate; *β*-sitosterol; *β*-D-glucopyranoside; and (1R)-*O*-isopropyl 6-*O*-(2,3,4-tri-*O*-acetyl-*β*-D-xylopyranosyl)-2,3,4-triacetate [9,11].

From the extracts of the leaves of *C. quercifolius*, it was possible to isolate the compounds linamarin and trans-cinnamic acid, as well as steroids and triterpenes [122]. From the extracts of the stem bark, it was possible to isolate the compounds lupeol-3*β-O*-cinnamate, lupeol-3*β* *O*-dihydrocinnamate, bis-nor-diterpene phylcanthone [123,124], lupeol-3*β*-*O*-cinnamate, lupeol-3*β-O*-dihydrocinnamate, lupeol-3*β-O*-hexanoate, bis-nor-diterpenes phylcanthone, and favelanone [125].

From the extracts of the leaves of *C. spinosus* it was possible to isolate the compounds 3-oxo-hop-22(29)-ene, 3*β*-hydroxy-hop-22(29)-ene, 3-oxo hop-22(29)-ene, and 3*β*-acetoxy-hop-22(29)-ene [126]. From the purification of fractions of the roots of *C. souzae*, it was possible to isolate the compound 7-deoxynimbidiol [127].

The species *C. aconitifolius, C. chayamansa,* and *C. quercifolius* have been the subject of numerous studies analyzing their chemical compositions, revealing their complexity and the variety of secondary metabolites produced by the species. However, most of these studies are associated with chemical analysis of leaves only, leaving aside the other parts of the plant that may be promising sources of chemical agents with therapeutic potential. From this perspective, it is necessary that new studies of the species analyze the chemical constituents present in the other parts of the plants in order to contribute to the discovery of new bioactive substances. Few studies were found addressing the chemical aspects of the species *C. urens*, *C. phyllacanthus*, *C. multilobus*, *C. aurifolia*, *C. spinosus*, *C. souzae*, and *C. texanus*; mainly these studies concerned isolated compounds, and more studies with this purpose are needed. From this perspective, a more detailed chemical analysis of these species is necessary in the search for new substances with medicinal or food applications and for the development of biotechnologies for sustainable use.

Table 2 presents the phenolic compounds, particularly flavonoids, that have been isolated and identified in various species of the genus *Cnidoscolus*. These secondary metabolites constitute the most prominent class identified in this study, exhibiting the strongest correlation with nutritional aspects, antioxidant activity, and other bioactivities discussed herein.

## 6. Biological Activities

### 6.1. Antioxidant Activity

Several studies have demonstrated the antioxidant potential of species of the *Cnidoscolus* genus, especially *C. aconitifolius*, whose leaves have been extensively investigated [36,37,55,82,128,129,130,131,132]. The antioxidant activity of this species has been investigated through various assays, and the study by Nnadiukwu and Nnadiukwu [77] indicated that the leaves of *C. aconitifolius* exhibit antioxidant activity, with superoxide dismutase values of 0.076 ± 0.004 Units/g, peroxidase activity of 0.442 ± 0.027 activity/min/u/g, and an IC_50_ of 12.95 for the DPPH^•^ radical (2,2-diphenyl-1-picrylhydrazyl), outperforming ascorbic acid (0.13). Additionally, the ethyl acetate fraction of the leaves demonstrated high antioxidant capacity with an IC_50_ of 23.11 μg/mL for metal ion chelation and an IC_50_ of 14.14 μg/mL for the ABTS^+^ radical (2,2′-azino-bis(3-ethylbenzothiazoline-6-sulfonic acid)) [10]

The research by Prajanban and Fangkrathok [133] revealed that immature, mature, and aged leaves of *C. aconitifolius* possess antioxidant activity, with aged leaves showing better results with an IC_50_ of 2.27 ± 0.02 μg/mL in the DPPH^•^ assay and an FRAP of 7.68 ± 0.15 mg GAE/g. In turn, Bulama et al. [134] observed through the DPPH^•^ and ABTS^+^ tests that the aqueous (IC_50_ values of 78.599 and 70.89 μg/mL, respectively) and dichloromethane fractions (IC_50_ values of 87.754 and 66.68 μg/mL, respectively) also presented good results.

Moreover, the aqueous leaf extract showed superiority over the methanolic extract, presenting an antioxidant capacity equivalent to 321.74 ± 2.4 μM/mL of Trolox, compared to 234.35 ± 27.1 μM/mL for methanol [135]. The study by Dangana, George, and Agboola [136] investigated the aqueous extract of *C. aconitifolius* and zinc oxide nanoparticles derived from this plant, confirming antioxidant activity in the H₂O₂ scavenging, DPPH^•^, and ABTS^+^ tests. Although inferior to ascorbic acid, the iron reducing activity (FRAP) of the extract was better than that of the nanoparticles at higher concentrations.

The antioxidant effect of *C. aconitifolius* in individuals with dyslipidemia was investigated by Guevara-Cruz et al. [44], and the results revealed a significant increase in plasma antioxidant activity and polyphenol concentration after 6 weeks of beverage consumption (*p* < 0.005). Antioxidant activity was quantified at 807 ± 10.53 Trolox equivalents (μmoles/L), and the oxidative stress marker malondialdehyde (MDA) showed a marked reduction (*p* < 0.0001), indicating an improvement in oxidative stress among participants.

The study by Rodrigues et al. [42] suggested that subcritical water extraction maximizes the antioxidant potential of the leaves, surpassing microwave-assisted and Soxhlet extraction methods. In a study conducted by Adefegha and Oboh [18], it was demonstrated that cooking increased the antioxidant activity of the leaves, due to an increase in phenol and flavonoid contents, in contrast to the findings of Kuti and Konuru [83], who observed better activity in raw leaves of *C. aconitifolius* and *C. chayamansa* compared to cooked ones, where the oxygen radical absorbance capacity (ORAC) values ranged from 15.6 to 11.8 μM TE/g in the extract of *C. aconitifolius* and from 15.6 to 14.8 μM TE/g in the extract of *C. chayamansa*.

The leaves of *C. chayamansa* were also investigated by other researchers. Aguirre Crespo et al. [86] observed activity in the DPPH^•^ assay (EC_50_ 49.6 ± 2.4 μg/mL) but no significant effect (>500 μg/mL) in the *β*-carotene assay. García-Rodríguez et al. [96] found no relevant antioxidant activity in the extracts of this species in the DPPH^•^ and FRAP tests, corroborating the findings of Loarca-Piña et al. [85], who reported high IC_50_ values (1693 ± 1.2 μg/mL) for ABTS^+^, indicating low antioxidant potency compared to controls (15.39 μg/mL for gallic acid and 43.90 μg/mL for Trolox). Relatively low inhibition capacity was also verified by Ramos-Gomez et al. [84] in the DPPH^•^ (IC_50_ 25.5 ± 2.8), ABTS^+^ (IC_50_ 38.5 ± 3.2), and NO (IC_50_ 44.3 ± 2.5) assays.

The impact of the drying method was analyzed by Hutasingh et al. [13], who found that cold drying better preserved the antioxidant compounds of *C. chayamansa*, with good results in the DPPH^•^ (691.50 ± 39.37 µmol TE/g dry weight) and FRAP (513.75 ± 29.53 µmol TE/g dry weight) assays. Antioxidant activity was also evaluated in cell culture extracts of *C. chayamansa* aimed at lupeol acetate production, with moderate results in the DPPH^•^ (EC_50_ 16.60 mg/mL) and ABTS^+^ (9.55% inhibition) assays [137].

Studies involving the seeds of *C. quercifolius* also showed significant antioxidant potential. Santos et al. [107] observed that the seed oil presented 76.68% DPPH^•^ radical scavenging, as well as an antioxidant capacity of 3.83 mmol Trolox/kg oil in the ABTS^+^ test. Meanwhile, Ribeiro et al. [19] reported that seed oil inhibited 32.20% of DPPH^•^ radicals at a concentration of 5 mg/mL and presented an IC_50_ of 52.45 ± 4.16 mg/mL in the ABTS^+^ radical assay.

Furthermore, Ribeiro et al. [113] observed that the pressed cake of the species reached 96.63 ± 1.62% inhibition in the DPPH^•^ assay, evidencing the superiority of the pressed cake compared to the seed (81.53 ± 1.80%). This result was confirmed in other studies by the same authors, where the pressed cake showed activity through the DPPH^•^ test (7.31 μM TE/g), reducing power (13.67 mgAA/g), total antioxidant activity (1.55 mgAA/g), ORAC (23.40 μM TE/g), and superoxide scavenging (21.86%) [67]. Moreover, they also observed that the flour of the pressed cake exhibited high potential, with values of 0.45 ± 0.00 and 42.83 ± 1.30 g TE/g for the DPPH^•^ and ABTS^+^ methods, respectively [73].

The DPPH^•^ method was also used to determine the antioxidant capacity of methanolic extracts of the roots, leaves, and root bark of *C. quercifolius*, which presented IC_50_ values of 171.82 ± 0.69, 133.30 ± 0.73, and 21.56 ± 0.71 µg/mL, respectively, demonstrating that the species, particularly the stem, is potentially effective at neutralizing free radicals [5]. In an earlier study on the various parts (leaves, branches, and roots), it was found that the leaves stood out, with an IC_50_ of 58.3 ppm [121]. However, when investigating the antioxidant potential of methanolic and ethyl acetate extracts of *C. quercifolius* barks, low potential was observed, such that it was impossible to calculate the IC_50_ at the tested concentrations (10 to 1000 µg/mL) [138].

A study conducted by Santos et al. [112] investigated the effects of alternative solvents on the antioxidant activity of *C. quercifolius* seed oil extracted with different solvents using the Soxhlet method, as well as pressurized ethanol extraction. According to the authors, the oil extracted with ethanol showed higher antioxidant activity in both the DPPH^•^ (6.0 mmol Trolox/kg oil) and ABTS^+^ (4.1 mmol Trolox/kg oil) assays. Oils extracted with n-hexane, isopropanol, and ethyl acetate showed lower antioxidant activities, with DPPH^•^ values of 4.1, 4.7, and 4.6 mmol Trolox/kg oil, respectively, and ABTS^+^ values of 3.3, 3.3, and 3.2 mmol Trolox/kg oil, respectively.

Moreover, the authors also indicated that CO_2_ supercritical extraction provided greater antioxidant activity, with values of 6.11 and 6.82 mmol Trolox/kg oil in the DPPH^•^ and ABTS^+^ assays, respectively [111]. Further studies on extraction methods also highlighted the superiority of ultrasound-assisted extraction, which provided oils with about 30% higher antioxidant activity than those extracted by Soxhlet [87]. Regarding the leaves of *C. quercifolius*, Torres et al. [139] identified a correlation between rutin extraction and antioxidant activity, with better results obtained at 45 °C and 1000 rpm, highlighting the importance of the extraction method in maximizing antioxidant potential.

Although studies on other *Cnidoscolus* species are less numerous, researchers have evaluated the antioxidant potential of different parts of these plants. The root extract of *C. souzae* was analyzed by Zapata-Estrella et al. [127] and showed significant antioxidant activity, with EC_50_ values of 1.58 ± 0.02 mg/mL. The semi-purified hexane fraction showed even higher activity (EC_50_ 0.75 ± 0.03 mg/mL), and the limonoid 7-deoxynimbidiol, with an EC_50_ of 0.60 μM, was identified as the main compound responsible for this antioxidant activity.

In contrast, the methanolic extract and polar fractions of *C. tehuacanensis* leaves showed limited antioxidant capacity, with IC_50_ values ranging from 0.5 to 4.01 mg/mL, values much higher than the quercetin standard, which presented an IC_50_ of 4 μg/mL. These results suggest that very high concentrations would be needed for effective inhibition of DPPH^•^ activity [117].

On the other hand, the leaf extract of *C. multilobus* showed antioxidant potential. Sánchez-Aguirre et al. [89] reported 84.2% inhibition of the DPPH^•^ radical, along with strong iron-reducing power, with 818.18 μmol of Fe^2+^/L, highlighting the antioxidant potential of this species. Additionally, Tinco-Jayo et al. [140] evaluated the spray-dried extracts of the leaves and stems of *C. diacanthus*. The stem extract outperformed in all the tests conducted, with values of 597.20 μmol/g (DPPH^•^), 452.67 μmol/g (ABTS^+^), and 535.91 μmol/g (FRAP). In comparison, the leaf extract showed lower values: 462.39 μmol/g (DPPH^•^), 202.32 μmol/g (ABTS^+^), and 198.13 μmol/g (FRAP), evidencing that the stem of this species has greater antioxidant activity than the leaves.

These results indicate that *Cnidoscolus* species, especially *C. aconitifolius* and *C. quercifolius*, demonstrated strong antioxidant activity in various tests, with variations depending on the extraction method, plant part type, and preparation conditions. However, while some species of the genus show good antioxidant potential, others require higher concentrations to exert significant effects. The identification of specific compounds, such as the limonoid 7-deoxynimbidiol in *C. souzae*, provides clues about those responsible for the antioxidant activity, suggesting that effectiveness can vary substantially among species and plant parts; nevertheless, further studies are still needed to fully understand their potential.

### 6.2. Antimicrobial Activity

Several studies have identified *C. quercifolius* as a possible source of new antibacterial agents. The antibacterial potential of extracts from stem bark and isolated compounds was evaluated against Gram-positive (*Enterococcus faecalis* and *Staphylococcus aureus*) and Gram-negative strains (*Escherichia coli*, *Klebsiella pneumoniae*, and *Serratia marcescens*). Isolated compounds such as phyllacanthone and a mixture of lupeol-3*β-O*-cinnamate and lupeol-3*β-O*-dihydrocinnamate exhibited significant bacteriostatic effects, with minimum inhibitory concentrations (MICs) of 0.25 and 0.5 mg/mL, respectively, surpassing hexane and methanolic extracts, which showed MICs of 5.0 mg/mL. Phyllacanthone also demonstrated notable bactericidal activity (MBC = 0.25 mg/mL), outperforming gentamicin against some strains, such as *E. faecalis* and *E. coli* [123].

More recent studies have highlighted the species’ potential against Gram-negative bacteria. Nascimento et al. [141] reported that methanolic and ethyl acetate extracts from the bark of *C. quercifolius* exhibited activity against standard and multidrug-resistant strains, such as extended-spectrum *β*-lactamase (ESBL)-producing *K. pneumoniae*, carbapenemase-producing *K. pneumoniae* (KPC), and *E. coli* ATCC 25922 [142], with MICs ranging from 256 to 512 μg/mL. The authors also reported synergistic effects between the extracts and antibiotics such as gentamicin, amoxicillin, and amikacin, except against KPC strains; synergy with penicillin was observed only against ESBL strains and *S. aureus* ATCC 29213 [142].

In another study, methanolic extracts from roots, leaves, and root bark of *C. quercifolius* showed good antibacterial activity, particularly against *Enterococcus faecium*, *Enterococcus faecalis*, *Staphylococcus epidermidis*, and *Pseudomonas aeruginosa* [5]. However, extracts obtained from seeds [67] and the inner bark [143] did not exhibit antibacterial activity. Ethanolic extracts from leaves, their partitioned fractions, and isolated compounds were also evaluated but showed no significant antibacterial properties (MIC > 1600 µg/mL), except for linamarin, which displayed moderate activity against *E. coli* (MIC of 1000 µg/mL) and weak activity against *S. aureus* and *P. aeruginosa* (MIC of 2000 µg/mL) [122]. Similarly, Alves et al. [144] observed that the hydroalcoholic extract of leaves did not exhibit significant antimicrobial activity (MIC > 2000 μg/mL).

On the other hand, extracts from *C. aconitifolius* showed promising antimicrobial results. Nanoparticles and extracts from this species were effective against *E. coli* and *Bacillus cereus*, with inhibition zones of 24.3 mm and 21 mm, respectively, at the highest tested concentrations (100 μg) [136]. The antimicrobial potential of the species was also confirmed against *S. aureus*, *P. aeruginosa*, and *Candida albicans* [128].

Furthermore, the dichloromethane–methanol extract of *C. aconitifolius* leaves was effective against *Helicobacter pylori* (MIC = 62.5 µg/mL), outperforming metronidazole, although the aqueous extract showed no activity against this bacterium [80]. Additionally, the ethanolic leaf extract did not demonstrate significant antimicrobial activity against multidrug-resistant strains (*E. coli*, *Klebsiella* spp., *S. aureus*, and *C. albicans*) [94]. Ethanolic extracts from the leaves and stems of *C. urens*, at concentrations ranging from 1:0 to 1:5, were shown to effectively inhibit the fungal mycelial growth of *Colletotrichum* spp. [120].

The antibacterial and resistance-modulating activities of ethanolic extracts from the leaves, stems, and roots of *C. urens* against multidrug-resistant bacteria were also evaluated by Oliveira et al. [145]. However, the MICs obtained for all extracts were greater than 1024 μg/mL. In terms of resistance modulation, the leaf extract in combination with gentamicin yielded the best results, significantly reducing the MICs of all tested bacteria, particularly *E. coli*, *Lactococcus garvieae*, and *Staphylococcus sciuri*. Moreover, combined with erythromycin, the extract reduced the MICs of *E. faecium*, *L. garvieae*, *S. aureus*, *Staphylococcus epidermidis*, and *Streptococcus agalactiae*.

The antimicrobial activity of *C. chayamansa* has also been investigated. Although its antibacterial activity was classified as moderate, the extract from a cell suspension culture of *C. chayamansa* was effective against *S. aureus*, *Listeria monocytogenes*, and coagulase-positive *Staphylococcus* at a concentration of 1 mg/mL [137]. Similarly, another study showed that the species was active against *S. aureus* [11]. Additionally, the organic leaf extract (CHCl_3_:MeOH) exhibited an MIC of 50 mg/mL against *Mycobacterium tuberculosis* and monoresistant and MDR strains of *M. tuberculosis*, with isolated compounds such as moretenol and moretenyl acetate displaying MICs of 25 mg/mL. However, isolated flavonoids, such as kaempferol-3,7-dimethyl ether and 5-hydroxy-7-3′,4′-trimethoxyflavanone, did not exhibit activity against *M. tuberculosis* (MICs > 50 mg/mL) [9].

Finally, the endogenous fluid from *C. multilobus* trichomes exhibited antimicrobial activity against *Pseudomonas syringae*, *Fusarium oxysporum*, and *Bemisia tabaci*, with MICs ranging from 97.4 to 122.2 µL/mL, but showed no effect against *Clavibacter michiganensis* [146]. Conversely, the extract of *C. tehuacanensis* did not show significant antibacterial activity against various strains [117].

Although numerous studies have demonstrated the antibacterial activity of *Cnidoscolus* species and their compounds, with particular emphasis on *C. quercifolius* and *C. aconitifolius*, important gaps remain. The antimicrobial activity of many extracts is moderate, and efficacy against multidrug-resistant strains varies depending on the extraction method and tested concentrations. Some extracts, such as those from seeds and leaves of *C. quercifolius*, showed no significant activity, whereas isolated compounds like phyllacanthone yielded promising results. Furthermore, the synergistic effects between extracts and antibiotics require further exploration to optimize antimicrobial therapies. The lack of activity against certain strains and variability in results highlight the need for deeper studies on the mechanisms of action and the toxicity of these compounds.

### 6.3. Anti-Inflammatory and Antinociceptive Activity

The extract and fractions from the leaves of *C. tehuacanensis* demonstrated significant anti-inflammatory activity, with an effective dose (ED_50_) of 1.79 mg/ear in the 12-*O*-tetradecanoylphorbol-phosphate (TPA)-induced edema test, a value similar to that of indomethacin (ED_50_ = 1.73 mg/ear). In the carrageenan-induced paw edema test, the ED_50_ was 567.3 mg/kg. Fractions containing lupeol acetate and *β*-amyrin acetate showed the best anti-inflammatory responses [117].

Research on *C. chayamansa* also indicated an anti-inflammatory effect, although with results inferior to the positive control (indomethacin = 50.06%). Hexane, ethyl acetate, and ethanol extracts showed edema reduction (30.76%, 32.04%, and 31.90%, respectively). The results are in line with the study conducted by Pérez-González et al. [9], which demonstrated that the species inhibited TPA-induced edema formation by 27.62%, 37.19%, and 55.46% at doses of 0.5, 1, and 2 mg/ear, respectively. Moreover, isolated compounds such as moretenol and kaempferol-3,7-dimethyl ether exhibited topical activity (30.34% and 31.84%, respectively) [9]. In a subsequent study, *C. chayamansa* demonstrated a dose-dependent effect in the carrageenan-induced paw edema model (ED_50_ = 351.53 and 50.11 mg/kg) and TPA-induced ear edema, with the extract and fraction presenting ED_50_ values of 1.58 and 1.48 mg/ear, respectively [105].

The seed oil of *C. quercifolius* also showed anti-inflammatory and antinociceptive efficacy, with edema inhibition superior to indomethacin at a concentration of 500 mg/kg, indicating not only anti-inflammatory but also antinociceptive potential [19]. Additionally, the bark and leaves of *C. quercifolius* (100, 200, and 400 mg/kg) significantly inhibited (*p* < 0.01) edema increase and leukocyte migration in the carrageenan model [106].

*C. souzae*, through the isolated compound 7-deoxynimbidiol, showed a significant reduction in carrageenan-induced paw edema, as well as antinociceptive activity against prostaglandin E2 and bradykinin [127]. *C. aconitifolius* exhibited both topical and systemic anti-inflammatory effects, with the ethyl acetate fraction from the leaves (25 and 50 mg/kg) reducing ear edema in a dose-dependent manner (23.52% and 49.41%, respectively) [78] and the ethanolic extract (100 or 200 mg/kg) inhibiting paw edema in inflammation models [147].

Nanoparticles and aqueous extracts from the leaves of *C. aconitifolius* exhibited anti-inflammatory and antinociceptive activity, with inhibition of red blood cell hemolysis in the membrane stabilization test (82.59% and 72.88%, respectively) and inhibition of proteins such as proteinase (95.93%) [136]. Moreover, phenolic and flavonoid fractions showed a reduction in the expression of pro-inflammatory proteins TNF-α (35% and 40%, respectively) and IL-6 (49% and 51%, respectively) in diabetic rats, suggesting a mechanism of action mediated by phenolic compounds, especially ferulic acid [148].

It was also observed that the methanolic extract of *C. aconitifolius* leaves demonstrated a significant protective effect against lipopolysaccharide (LPS)-induced neuroinflammation in rats. In this study, extract administration also reduced TNF-α and IL-6 levels in the prefrontal cortex and hippocampal region of the rats. It was suggested that the anti-inflammatory properties of this extract reflect its ability to suppress microglial activation and astrogliosis in the brain [82].

Finally, isolated compounds from the leaves of *C. spinosus*, such as 3*β*-acetoxy-hop-22(29)-ene, 3-oxo-hop-22(29)-ene, and 3*β*-hydroxy-hop-22(29)-ene, also exhibited anti-inflammatory action through the TPA-induced edema model. Notably, 3*β*-acetoxy-hop-22(29)-ene was the most active in the TPA-induced edema model, reducing inflammation by 57.27% and presenting an ID_50_ of 0.36 μmol/ear. According to the authors, the spatial orientation of the isopropenyl group at C-21 in pentacyclic triterpenes is an important characteristic for the anti-inflammatory activity of the compounds analyzed [126].

Although the exact mechanism has not been detailed in the studies, the anti-inflammatory activity has been attributed by some authors to the phenolic compounds present in the extracts, which are known to mediate the inflammatory response. However, despite studies revealing significant therapeutic potential for these species, some gaps remain, such as the lack of investigations into the mechanisms of action of the extracts and bioactive compounds. Furthermore, expanding research using different experimental models and evaluating side effects are essential to validate the therapeutic applications of these plants, particularly in drug development.

### 6.4. Hypoglycemic Activity

Several studies suggest that species of the genus *Cnidoscolus*, especially *C. aconitifolius* and *C. quercifolius*, show potential in glycemic control, indicating a possible application as complementary treatments in diabetes management. In the study by Mahammad et al. [149], the combination of metformin with teas from *C. aconitifolius* in rats with type 2 diabetes resulted in a significant reduction (*p* < 0.05) in fasting blood glucose, triglycerides, and LDL cholesterol levels, in addition to improvements in liver and kidney function markers, such as ALT, AST, creatinine, and urea. The results also demonstrated that the combination of teas with metformin was more effective than metformin alone in controlling fasting glycemia, with the best results observed in the group that received green tea. This study reinforces the idea that combining natural and pharmacological therapies may be more effective than using conventional medications alone.

Roy et al. [12] and Achi et al. [150] observed a reduction (*p* < 0.05) in glucose levels in streptozotocin-induced diabetic mice after administration of the ethanolic extract of the plant. The study by Roy et al. [12] evidenced a dose-dependent reduction in glucose levels, with a significant reduction at a dose of 200 mg/kg (from 245.33 ± 13.01 mg/dl to 187.03 ± 7.19 mg/dl), while Achi et al. [150] demonstrated a drop in glycemic levels after glucose loading, suggesting that bioactive compounds such as tannins and saponins may be responsible for these hypoglycemic properties.

Additionally, Manzanilla Valdez et al. [151] investigated several extracts of *C. aconitifolius* (aqueous, ethyl, acetonic, ethyl acetate, diethyl ether, and hexane) and found that the hexane extract showed the best hypoglycemic effect, with a reduction of up to 22.9% in blood glucose levels. The inhibition of α-amylase and α-glucosidase enzymes, responsible for carbohydrate digestion, was also suggested as a mechanism of action, with the ethyl extract demonstrating the lowest IC_50_ values (22.97 and 3.20 μg/mL, respectively) for these enzymes.

Moreover, the hypoglycemic potential of the aqueous leaf extract of *C. aconitifolius* was also evidenced by Ajiboye et al. [36]. According to the results of this study, the extract is capable of improving glucose uptake under ex vivo conditions, suggesting a hypoglycemic effect, attributed to its ability to inhibit oxidative stress, a contributing factor to hyperglycemia in type 2 diabetes.

Further studies indicate ameliorative effects of *C. aconitifolius* on several health parameters in alloxan-induced diabetic rats. After treatment with the ethanolic extract, notable increases were observed in packed cell volume (PCV), red blood cell count (RBC), hemoglobin levels (Hb), white blood cell count (WBC), and mean corpuscular volume (MCV). These improvements suggest that the extract may enhance erythropoiesis, combating the anemia often associated with diabetes. Additionally, the extract also demonstrated an ameliorative effect on sperm quality, showing improvements in sperm count, motility, and the live/dead ratio, which is particularly important given the fertility reduction caused by diabetes [40].

Regarding the activity on digestive enzymes and glycemic control, Ajiboye et al. [10] demonstrated that the ethyl acetate fraction of *C. aconitifolius* leaves was effective in inhibiting α-amylase (IC_50_: 13.85 μg/mL) and α-glucosidase (IC_50_: 18.98 μg/mL), crucial enzymes in carbohydrate metabolism, suggesting a potential role of the plant in diabetes management. Furthermore, in a more recent study, Calonico and De La Rosa-Millan [131] found that *C. aconitifolius* also inhibits several digestive enzymes, such as α-amylase (IC_50_: 471.3 mg/mL), α-glucosidase (IC_50_: 433.78 mg/mL), lipase (IC_50_: 627.84 mg/mL), and pepsin (IC_50_: 1039.93 mg/mL). Although the IC_50_ values in this study are relatively high, they indicate significant inhibitory action that may contribute to glycemic and obesity management.

These findings were reinforced by López-Huerta et al. [126], who identified the compound 3*β*-acetoxy-hop-22(29)-ene, isolated from the leaves of *C. spinosus*, as an effective inhibitor of α-glucosidase in tests performed with both yeast and rats. The observed effect was attributed to the chemical structure of the compound, which enables favorable interaction with the enzyme’s active site.

Studies on *C. quercifolius* also demonstrate hypoglycemic potential. Lira et al. [109] reported that the aqueous leaf extract reduced blood glucose levels by 29% in mice, without causing alterations in organs such as the liver, pancreas, and kidneys. According to the authors, this effect may be related to the presence of flavonoids, catechins, and triterpenoids in the plant.

The protein Cq-IMP, isolated from *C. quercifolius* seeds, was also investigated for its hypoglycemic activity. Moura et al. [4] reported that Cq-IMP showed a lasting hypoglycemic effect, with concentration-dependent reduction and no cellular toxicity. These results indicate promising therapeutic potential for type 1 and 2 diabetes, suggesting that future research should focus on the clinical application of this protein.

Another species that has shown potential hypoglycemic activity in preclinical models is *C. chayamansa* [81]. The methanolic extract of its leaves, at a dose of 70 mg/kg, caused a significant reduction in blood glucose levels in diabetic rats, with effects similar to those of glibenclamide [85]. The authors suggested that the hypoglycemic action of *C. chayamansa* may be mediated by stimulating insulin secretion by the remaining beta cells in the pancreases of rats with moderate diabetes. However, Ramos-Gomez et al. [84] observed that this effect is not related to insulin secretion but rather to the decrease in glucose absorption, indicating the need for more studies to elucidate the mechanisms involved.

In the study conducted by Cárdenas-Ibarra et al. [152], the potential of *C. chayamansa* in reducing hyperglycemia in women with early metabolic syndrome (EMS) was evaluated. However, despite positive reports from participants regarding improvements in general well-being and energy levels, the ingestion of infusions prepared from dehydrated leaves of the plant did not result in a significant reduction in glycemic levels, HDL, or triglycerides. The authors attributed the absence of relevant therapeutic effects possibly to the preparation method (microwave dehydration), which may have compromised essential bioactive compounds responsible for the hypoglycemic action.

These studies suggest that species of the genus *Cnidoscolus* may have potential as adjuvants in diabetes management, particularly in glycemic control, based on preliminary or preclinical evidence. However, there is a lack of robustly designed in vivo and in vitro studies investigating the mechanism of action of the plant on glucose metabolism. Therefore, further research is still needed to isolate and characterize the bioactive compounds responsible for these effects, as well as to investigate the mechanisms of action involved. This will allow for a better understanding of the therapeutic potential of these plants and their possible clinical applications as alternatives to traditional medications, which often present undesirable side effects.

### 6.5. Hepatoprotective Activity

Based on the studies analyzed, it was observed that *C. chayamansa* and *C. aconitifolius* have shown promising results regarding hepatoprotective activity in different models of hepatotoxicity. Pérez-González et al. [11] evaluated the hepatoprotective activity of *C. chayamansa* against hepatotoxicity induced by antitubercular drugs. The leaf extract improved body weight gain and reduced liver enzyme levels (AST and ALT), in addition to attenuating oxidative stress, evidenced by the decrease in parameters such as SOD, CAT, Lpx, and POx. Histologically, the absence of steatosis was observed in the group treated with 400 mg/kg of the extract, whereas the groups treated with silymarin and 200 mg/kg showed moderate steatosis.

The CHCl_3_:MeOH extract of *C. chayamansa* leaves also demonstrated a hepatoprotective effect in animals with liver damage induced by anti-TB drugs [105]. Additionally, it was confirmed that the aqueous extract of its leaves can reduce phenylhydrazine-induced toxicity by decreasing liver enzymes, including alanine aminotransferase and aspartate aminotransferase [47].

Regarding *C. aconitifolius*, the study by Oyagbemi and Odetola [153] showed that the ethanolic leaf extract had a significant hepatoprotective effect in rats subjected to paracetamol intoxication, with a dose-dependent effect. The 1000 mg/kg dose was the most effective, resulting in a significant reduction (*p* < 0.05) in liver enzyme levels (ALT, AST, and ALP). In contrast, the study by Ezebuiro et al. [154] found that the administration of the hydromethanolic leaf extract did not cause significant effects on liver enzymes (AST, ALT, and ALP) or on hepatic and renal functions when administered at doses of 200 and 400 mg/kg. This suggests that the extract administration did not cause toxic effects and that it is effective as a hepatoprotective and renoprotective agent. According to the authors, the hepatoprotective potential may be related to the presence of flavonoids, which help stabilize hepatic cell membranes.

Moreover, the study by Somade et al. [96] reported that the leaf extract of *C. aconitifolius* (400 mg/kg) showed a protective effect against dimethylnitrosamine (DMN)-induced hepatotoxicity in animal models. Histopathological analyses indicated fewer liver lesions in the group treated with the extract, demonstrating that the treatment helped mitigate DMN toxicity. The authors suggest that the hepatoprotective activity may be related to the extract’s antioxidant capacity, which reduces the generation of reactive oxygen species (ROS) during DMN metabolism.

Additional studies have also evidenced the efficacy of *C. aconitifolius* in preventing liver damage. Oboh [155] observed that a diet supplemented with *C. aconitifolius* in rats fed garlic significantly reduced (by 40%) hepatic transaminase levels (SGOT and SGPT), indicating a protective effect against garlic-induced hepatotoxicity. The species’ antioxidant potential was also cited as responsible for its hepatoprotective effect. Furthermore, Ikewuchi et al. [156] confirmed that the aqueous leaf extract of *C. aconitifolius* demonstrated a protective effect against doxorubicin-induced hepatorenal toxicity.

Another study by Oyagbemi and Odetola [41] showed that during recovery, the inclusion of *C. aconitifolius* in the diets of malnourished rats resulted in a significant reduction in liver enzyme levels (ALP, ALT, and AST), blood urea, and creatinine, in addition to an improvement in renal function, suggesting that the plant may play a role in the recovery from liver and kidney damage caused by protein–energy malnutrition.

The available studies suggest that *C. chayamansa* and *C. aconitifolius* may exhibit hepatoprotective properties in various experimental models of liver injury. Although the presence of flavonoids in *C. aconitifolius* has been associated with these protective effects, further research is needed to elucidate the molecular mechanisms involved and to identify the specific active compounds. Moreover, comparing different types of extracts and dosages, such as ethanolic, hydromethanolic, and aqueous formulations, could contribute to optimizing extract composition and clarifying discrepancies observed across studies.

It is also important to emphasize the need for comprehensive studies on lesser-known species within the *Cnidoscolus* genus, as current research has primarily focused on a limited number of well-characterized species. Given the structural and phytochemical similarities observed among various members of this genus, it is plausible that other species may also exhibit hepatoprotective—or other biologically relevant—properties. Investigating these underexplored species could not only broaden the pharmacological understanding of the genus but also uncover novel bioactive compounds with therapeutic potential. Furthermore, such studies are essential to address existing knowledge gaps, support biodiversity-based drug discovery, and guide the development of standardized, safe, and effective plant-based interventions.

### 6.6. Hypolipidemic Activity

Studies have evidenced the beneficial effects of species from the *Cnidoscolus* genus, especially *C. chayamansa* and *C. aconitifolius*, on lipid profiles in animal models with induced hyperlipidemia. The study conducted by Iswari et al. [45] demonstrated that treatment with the ethanolic leaf extract of *C. chayamansa* (100, 200, and 400 mg/kg) promoted a significant reduction in total cholesterol, triglyceride, and LDL-C levels in hyperlipidemic rats. The highest dose tested (400 mg/kg) resulted in expressive reductions, with total cholesterol at 76.81 ± 1.10 mg/dL, triglycerides at 72.39 ± 1.66 mg/dL, and LDL-C at 21.47 ± 0.58 mg/dL. The authors associated this activity with the presence of flavonoids with antioxidant and anti-inflammatory properties, which contribute to improved lipid metabolism and may act in the prevention of cardiovascular diseases such as atherosclerosis.

Complementing these findings, Miranda-Velasquez et al. [157] investigated ethanolic, methanolic, and aqueous leaf extracts of *C. chayamansa* in Balb/c mice with induced hyperlipidemia. Only the aqueous extract, at doses of 50 and 100 mg/kg, promoted significant reductions in cholesterol levels (27.9% and 31.1%, respectively; *p* < 0.01), while the organic extracts did not show a relevant effect. Interestingly, the aqueous extract did not inhibit the HMG-CoA reductase enzyme, suggesting that the present compounds act on distinct metabolic pathways. The authors hypothesized that alkaloids might be involved in the hypocholesterolemia mechanism.

Similarly, Sarsanti et al. [158] observed dose-dependent reductions in triglyceride levels in rats treated with the aqueous leaf extract of *C. aconitifolius* (150, 300, and 450 mg/kg), with statistical significance (*p* < 0.05). Additionally, an increase in body weight gain was reported in the treated groups. These effects were attributed to the presence of saponins and phenolic compounds, which are capable of inhibiting lipase activity and modulating hepatic lipid metabolism.

Despite the promising results, some scientific gaps remain. The difference between the effects of aqueous and organic extracts observed by Miranda-Velasquez et al. [157] highlights the need for comparative studies between different extraction methods and extract preparations to enable precise identification of the active compounds and their mechanisms of action. Furthermore, the absence of detailed mechanistic tests, such as evaluation of molecular signaling pathways, gene expression, and bioavailability studies, limits the understanding of the specific roles of flavonoids, alkaloids, saponins, and other secondary metabolites.

### 6.7. Antianemic and Antisickling Activity

In a study conducted by Atata et al. [38], the ethanolic leaf extract of *C. aconitifolius*, administered at doses of 100 and 500 mg/kg, significantly stimulated hematopoiesis, with emphasis on the erythroid lineage of the bone marrow. A dose-dependent increase was observed in hematological parameters, such as packed cell volume and hemoglobin levels, in rats with cyclophosphamide-induced anemia. These effects were attributed to the presence of phytonutrients, such as flavonoids and tannins, compounds known for their antioxidant properties and their contribution to the stabilization of erythrocyte membranes.

Similar findings were reported by Oyagbemi et al. [40], who observed hematopoietic activity associated with *C. aconitifolius* leaves. In the study, a diet incorporating this plant combined with soy protein promoted a reduction in the osmotic fragility of erythrocytes, suggesting potential improvements in red blood cell stability and longevity under experimental conditions.

Ezeigwe et al. [130] also observed a significant antianemic effect with the aqueous extract of *C. aconitifolius* in rats with phenylhydrazine-induced anemia, evidenced by increased hemoglobin levels and erythrocyte counts. Subsequently, the same authors [46] found that the combined administration of *C. aconitifolius* and *Ficus capensis* resulted in a synergistic effect superior to isolated use, with remarkable improvements in hemoglobin levels, hematocrit, and red blood cell counts (*p* < 0.05). Although the specific mechanism of action has not yet been elucidated, the authors suggest that phenolic compounds and flavonoids may be involved in the modulation of hematopoiesis.

Regarding antisickling activity, Cyril-Olutayo et al. [103] evaluated the ethanolic extract of *C. aconitifolius* and its fractions (n-hexane, dichloromethane, ethyl acetate, and methanol) in assays of inhibition and reversal of erythrocyte sickling. The crude extract demonstrated 80.4 ± 0.15% inhibition and 56.0 ± 2.90% reversal at 4 mg/mL, while the ethyl acetate fraction showed the best performance, with 68.0 ± 4.32% inhibition and 61.4 ± 6.2% reversal, demonstrating a similar effect to the positive control (Ciklavit = 59.8 ± 0.3% inhibitory and 56.6 ± 0.2% reversal). In this study, the activity was attributed to the compound tetramethylbicosahydropicen-3-ol, although additional assays are necessary for confirmation and elucidation of the mechanism of action.

Additionally, Kutshik et al. [47] investigated the antianemic effect of the aqueous extract of *C. chayamansa*, observing a significant increase (*p* < 0.05) in hematological parameters such as packed cell volume, hemoglobin, and red and white blood cell counts in albino rats with phenylhydrazine-induced anemia. The authors suggest that these effects may be associated with bioactive compounds present in the leaves that are capable of stimulating erythropoiesis or enhancing iron absorption.

From the studies analyzed, it was observed that the hematopoietic effects in the *Cnidoscolus* genus have been investigated exclusively in the species *C. aconitifolius* and *C. chayamansa*, whose experimental studies demonstrate promising therapeutic potential. Based on these studies, these species possess important therapeutic potential as antianemic and antisickling agents, contributing to the restoration of hematological parameters and the improvement of erythrocyte stability, which reinforces their value as adjuvants in functional nutrition and the management of hematological conditions, especially in contexts of malnutrition or chronic diseases. However, gaps still exist regarding the potential of these species, particularly concerning the lack of in-depth mechanistic studies, especially regarding the mode of action of active compounds, as well as controlled clinical trials in humans to validate the therapeutic efficacy observed in animal models.

### 6.8. Neuropharmacological Properties

Current research on the neuropharmacological effects of *Cnidoscolus* species is limited and largely restricted to early-stage experimental studies. Preliminary evidence indicates possible activity related to central nervous system function, including models of mood disorders, anxiety, and cognitive deficits.

The study conducted by Iyare et al. [43] revealed that the methanolic extract of *C. aconitifolius* leaves exhibits antidepressant, anxiolytic, and sedative–hypnotic activities in murine models. Administration of the extract significantly reduced immobility times in the forced swim test (*p* < 0.05) and tail suspension test (*p* < 0.05), suggesting an antidepressant effect. In the phenobarbital-induced sleep test, doses of 200 and 400 mg/kg decreased sleep latency time (*p* < 0.001) and prolonged sleep duration (*p* < 0.01), characterizing a sedative effect. In the elevated plus maze test, the 400 mg/kg dose increased the number of entries into the open arms, indicating a possible anxiolytic effect. Importantly, no anticonvulsant effects, muscle relaxant effects, or motor coordination alterations were observed, suggesting a favorable neurological safety profile.

Complementing these observations, Babalola et al. [82] demonstrated that the methanolic extract of *C. aconitifolius* leaves significantly improved memory and cognitive function in rats subjected to lipopolysaccharide-induced neuroinflammation. Y-maze and novel object recognition tests showed better cognitive performance in treated animals, which the authors attributed to possible inhibition of the acetylcholinesterase enzyme, responsible for the degradation of acetylcholine, a neurotransmitter essential for memory and learning processes. According to the researchers, these effects may be related to the modulation of oxidative stress and inflammatory processes in the central nervous system.

Additionally, Nascimento et al. [138] investigated the anxiolytic effects of the methanolic and ethyl acetate extracts of *C. quercifolius* bark in zebrafish (*Danio rerio*). According to the results, the extracts reduced locomotor activity at all tested doses (40, 200, and 400 mg/kg) and increased the time spent by the animals in the light zone, behavior consistent with an anxiolytic effect.

Despite the promising results, the studies are still exploratory and are mostly concentrated on *C. aconitifolius*, with few investigations involving other species of the genus. This limitation highlights the need to expand the scope to other species to verify whether the neuropharmacological effects are shared or species-specific. Moreover, mechanistic studies exploring the molecular pathways involved in the observed effects, such as the modulation of neurotransmitters (GABA, serotonin, and dopamine) or the influence on neuroinflammation and oxidative stress markers (NF-κB, BDNF, and IL-1*β*), are still lacking.

In this context, the importance of additional assays is emphasized, including complementary behavioral tests and biochemical analyses, as well as in-depth phytochemical investigations to isolate and identify the neuroactive compounds responsible for the observed effects.

### 6.9. Cardioprotective Activity and Cardiorenal Effects

Experimental studies indicate that species of the *Cnidoscolus* genus exhibit protective effects on the cardiovascular and renal systems, suggesting their therapeutic potential in the prevention and management of disorders associated with hypertension, renal dysfunction, and ischemic damage. In an ischemia/reperfusion model in rats, García-Rodríguez et al. [95] demonstrated that the ethyl extract of *C. chayamansa* leaves, administered orally at a dose of 500 mg/kg, conferred significant protection to cardiac tissue, suggesting a protective action against oxidative stress and ischemic injury. The authors highlight that such effects may be useful in the prevention or treatment of cardiovascular diseases.

In turn, Alawode et al. [159] reported that the ethanolic extract of *C. aconitifolius* promoted hypotensive and nephroprotective effects in hypertensive rats, with a reduction in systolic, diastolic, and mean arterial pressure, attributed to increased sodium excretion, a mechanism associated with the presence of flavonoids with diuretic properties. Additionally, an increase in creatinine and sodium excretion and regenerative effects in cardiac and renal tissues were observed, indicating protective action against hypertension-induced damage. Complementing these findings, Ezeigwe et al. [130] also evidenced the normalization of serum urea and creatinine levels in rats treated with the aqueous extract of *C. aconitifolius*, highlighting the role of the plant’s antioxidant compounds in reducing oxidative stress and inflammation in the kidneys.

However, when investigating the vasorelaxant potential of the methanolic extract of *C. chayamansa* in isolated rat aortic rings, Aguirre Crespo et al. [86] observed no significant vasodilatory effect even at high concentrations (>500 μg/mL). This finding suggests that, unlike *C. aconitifolius*, the cardiovascular effects of *C. chayamansa* do not directly involve the modulation of vascular tone through arterial smooth muscle relaxation, thus requiring further studies to identify its mechanisms of action.

Despite the advances, gaps remain regarding the mechanisms related to these cardiorenal effects, and studies are still limited to animal models. Thus, to achieve a better understanding of the therapeutic potential of these species, additional studies are still necessary, including detailed analysis of bioactive compounds by LC-MS/MS or NMR, functional assays on ion channels and vascular receptors, as well as experimental models with different etiologies of cardiovascular dysfunction, in addition to clinical trials to validate the therapeutic efficacy of these plants in humans.

### 6.10. Antiproliferative Activity and Cytotoxic

Several species of the genus *Cnidoscolus* have been extensively investigated for their antiproliferative and cytotoxic potential against various tumor and non-tumor cell lines. Overall, studies indicate that the effects of these plants vary considerably depending on the species, the type of extract, the fraction used, and the concentration applied.

In a study conducted by Sánchez-Aguirre et al. [89], the extract of *C. multilobus* demonstrated significant antiproliferative activity against the HeLa cell line (cervical cancer), with inhibition of cell proliferation at 125 μg/mL, as assessed by the MTT assay. However, concentrations above 250 μg/mL showed cytotoxic effects, suggesting that its therapeutic effects are dose-sensitive, being effective at specific concentrations but potentially toxic at higher doses.

In *C. aconitifolius,* both immature and mature leaves exhibited low cytotoxicity against RAW 264.7 macrophages (50–800 µg/mL), with a slight increase in toxicity observed only in senescent leaves at the highest concentration tested (800 µg/mL) [133]. However, *C. aconitifolius* demonstrated antiproliferative effects against human colon adenocarcinoma cells (SW480), promoting apoptosis and cell cycle arrest at the G0/G1 phase (CL_50_ = 10.65 mg/mL), as well as 63.08% inhibition of breast cancer (MCF-7) cell growth at 50 µg/mL [76]. At higher concentrations (200–250 µg/mL), cytotoxicity was more pronounced, reducing cell viability by −14.70% and −26.25%. Extracts from the stem and root were inactive, whereas the chloroform fraction exhibited high cytotoxic activity (GI_50_ values of 22.5 µg/mL for MCF-7 and 35.4 µg/mL for lung cancer cells (NCI-H460)). The aqueous fraction showed no significant effect [92].

In vivo studies also indicate chemopreventive potential. Kuri-García et al. [129] observed that an infusion of *C. aconitifolius* leaves effected a significant reduction in aberrant crypt foci (by 29.5% in the subchronic and 64.6% in the chronic phase) in a murine model of colon carcinogenesis induced by azoxymethane and dextran sulfate sodium, as well as attenuation of histopathological lesions in the colon. These effects were attributed to the presence of phenolic compounds with synergistic anti-inflammatory and antiproliferative properties.

In *C. quercifolius*, compounds isolated from the leaves, including linamarin and lupeol derivatives, were evaluated for their ability to inhibit the growth of exponentially growing human tumor cells but did not show antiproliferative effects at the tested concentrations [122]. On the other hand, the crude leaf extract of this same species exhibited nonspecific cytotoxic activity against various tumor cell lines, with an average GI_50_ (50% inhibitory concentration) value of 2.4 μg/mL, as demonstrated by Alves et al. [144], suggesting that the antiproliferative effects may be related to a set of compounds present in the total extract rather than isolated components. The ethyl acetate fraction of the leaves displayed cytotoxic activity against prostate (PC3 and PC3-M) and breast (MCF-7) cancer cell lines, with IC_50_ values ranging from 15.75 to 46.97 µg/mL [108]. Meanwhile, the chloroform fraction of the root bark was active against colon (HCT-116), ovary (OVCAR-8), and glioblastoma (SF-295) cell lines at 25 µg/mL [114]. On the other hand, the seed oil did not show significant cytotoxicity in RAW 264.7 macrophages, even at high concentrations (up to 5.000 µg/mL), indicating a favorable safety profile in non-tumor cells [19].

Special attention should be given to the compound phyllacanthone, isolated from the bark of *C. quercifolius*, which demonstrated cytotoxic effects against A2058 melanoma cells (BRAF-mutated), with an IC_50_ of 40.9 µM. Docking studies indicated strong interaction with tubulin at the colchicine binding site, suggesting a possible antimelanoma mechanism of action [125] These data were corroborated by Nai et al. [116], who also reported antimelanoma activity of phyllacanthone (IC_50_ 58.63 µM), as well as of the purified compounds deoxofavelin and favelin (IC_50_ = 9.67 and 11.63 µM, respectively) against A2058 cells.

Rocha et al. [124] showed that the nanoencapsulated form of phyllacanthone (Fe_3_O_4_@*β*CD-PHY) exhibited higher cytotoxicity than the free form in lung cancer cells (H292), without affecting normal VERO cells at concentrations up to 100 µM, indicating selectivity and enhanced bioactivity through nanotechnology. Additionally, Alves et al. [160] demonstrated that inclusion complexes of phyllacanthone with *β*-cyclodextrin and sulfobutylether-*β*-cyclodextrin increased the inhibition of A2058 cell growth to 42.98% and 39.95%, respectively. Molecular docking confirmed stable interactions with favorable binding energies (−89.81 and −87.40 kcal/mol), indicating the greater stability and solubility of the complexes.

Conversely, the triterpenes 3*β*-acetoxy-hop-22(29)-ene, 3-oxo-hop-22(29)-ene, and 3*β*-hydroxy-hop-22(29)-ene, isolated from the leaves of *C. spinosus,* did not exhibit antiproliferative effects against six human cell lines, including colon (HCT-15), leukemia (K562), glioblastoma (U251), breast (MCF-7), prostate (PC-3), and lung (SKLU-1) cell lines [126]. In *C. chayamansa*, the ethanolic leaf extract exhibited moderate cytotoxic activity against tumor (HT-29 colon carcinoma) and normal cell lines, with a dose-dependent and non-selective effect, reflected by similar CTC_50_ values regardless of cell origin [161].

Despite the promising results, the heterogeneity of the effects of crude extracts and isolated compounds suggests that the observed bioactivity is likely related to the combined action of various secondary metabolites—such as flavonoids, terpenoids, and phenolic compounds. Most studies focus on in vitro screenings, highlighting a lack of data on systemic toxicity, pharmacodynamics, and pharmacokinetics, which limits the assessment of the true therapeutic potential of these substances. Therefore, further studies are essential to elucidate the molecular mechanisms involved, including pathways related to the cell cycle, apoptosis (caspase-3, p53, and Bcl-2), oxidative stress, and inflammation. The application of omics approaches (such as transcriptomics and proteomics), as well as in vivo assays with dose–response designs and comprehensive toxicological evaluations, will be crucial to validate the efficacy and safety of these species as anticancer agents.

### 6.11. Inhibition of Neurological Enzymes

Species of the *Cnidoscolus* genus have also stood out for their potential in inhibiting key enzymes involved in neurological and metabolic disorders. Recent studies highlight the potential of these species as promising sources of natural compounds with therapeutic applications in diseases such as Alzheimer’s, diabetes, and hypertension.

Paredes et al. [5] observed that methanolic extracts from the leaves, roots, and root bark of *C. quercifolius* exhibited promising inhibition of the enzyme acetylcholinesterase (AChE), with inhibition halos ranging between 7 and 8 mm. These findings indicate the possible usefulness of the species in the treatment of neurological disorders such as Alzheimer’s disease, whose therapy often involves AChE inhibition.

Corroborating this potential, Babalola et al. [82] demonstrated that the methanolic extract of *C. aconitifolius* leaves promoted a significant reduction in AChE activity in the prefrontal cortex (PFC) and hippocampus (HPC) of rats, especially at a dose of 100 mg/kg. Enzyme levels dropped from 17.51 ± 0.24 to 11.82 ± 0.46 µM/min/mg protein in the PFC and from 20.51 ± 0.83 to 15.69 ± 0.53 µM/min/mg in the HPC. These results suggest a beneficial modulation of the cholinergic pathway, potentially favoring cognitive and memory functions impaired by neuroinflammatory processes.

Additionally, Ajiboye et al. [10] verified that the ethyl acetate fraction from *C. aconitifolius* leaves significantly inhibited AChE (IC_50_: 61.13 μg/mL) and butyrylcholinesterase (BChE) (IC_50_: 56.25 μg/mL), suggesting potential use in cholinergic disorders and neurodegenerative diseases. The authors also highlighted additional inhibitory effects on monoamine oxidase (MAO) (IC_50_: 2.56 μg/mL), tyrosinase and arginase (IC_50_: 27.56 μg/mL), as well as on ecto-5′-nucleotidase (IC_50_: 1.57 μg/mL), phosphodiesterase-5 (IC_50_: 22.51 μg/mL), and angiotensin-converting enzyme (ACE) (IC_50_: 56.33 μg/mL). All these activities were dose-dependent, indicating that *C. aconitifolius* possesses a broad spectrum of action on enzymes related to neurotransmission, blood pressure regulation, vasodilation, purinergic metabolism, and pigmentation, reinforcing its therapeutic potential in multiple pathologies, neurological disorders, and pigmentary disorders.

### 6.12. Antiprotozoa and Antiparasitic Activity and Effects on Invertebrates

Although with significant variations among the species and compounds analyzed, studies have shown that extracts and isolated compounds from species of the *Cnidoscolus* genus possess relevant biological activities against protozoa, parasites, and arthropods, with potential therapeutic and agricultural applications.

The leaf extract of *C. chayamansa* exhibited moderate antiprotozoal activity with IC_50_ values of 65.29 mg/mL against *Giardia lamblia* and 42.69 mg/mL against *Entamoeba histolytica*. Isolated compounds such as moretenol and moretenyl acetate were more potent against *E. histolytica* (IC_50_ values of 26.47 and 41.90 mg/mL, respectively), while flavonoids such as kaempferol-3,7-dimethyl ether and 5-hydroxy-7,3′,4′-trimethoxyflavanone exhibited greater activity against both protozoa, standing out with IC_50_ values between 24.88 and 27.43 mg/mL [9]. These data reinforce the therapeutic potential of secondary metabolites present in the species.

Conversely, the triterpenes 3-oxo-hop-22(29)-ene and 3*β*-hydroxy-hop-22(29)-ene, isolated from *C. spinosus*, exhibited only marginal antiparasitic activity against *Trypanosoma cruzi* and *Leishmania mexicana*, indicating the need for structural modifications or formulations to enhance their efficacy [126]. Similarly, linamarin, extracted from the leaves of *C. quercifolius*, did not demonstrate significant activity against *Leishmania braziliensis* at any of the tested concentrations [122].

Regarding acaricidal activity, the ethanolic leaf extract of *C. aconitifolius* demonstrated low efficacy against the immature stages (eggs, larvae, and nymphs) of the mite *Tetranychus urticae* but caused high mortality (83%) in adults and significantly reduced female fecundity [98]. Previous studies by Numa et al. [162] corroborate these findings, indicating a dose-dependent effect on fertility reduction and increased adult female mortality, suggesting its potential use in integrated pest management.

Additionally, the leaves of *C. aconitifolius* exhibited biostimulant properties when used as a source for the synthesis of zinc oxide nanoparticles. These nanoparticles, when applied to *Sorghum bicolor*, significantly increased leaf length and chlorophyll content, highlighting the potential of the extract as a nanofertilizer [163].

In the agricultural context, *C. urens* showed larvicidal activity against *Ascia monuste orseis* (the cabbage caterpillar). Ethanolic extracts from leaves and roots, as well as aqueous extracts from roots, prolonged the larval phase, reduced pupal viability and mass, and caused deformities in adults. A deterrent effect was also observed, except with the ethanolic extract from the stem. These effects were possibly attributable to the presence of terpenes in the extracts [164].

The variability in efficacy results suggests that the observed effects strongly depend on the type of extract, the plant part used, and the concentration tested, reinforcing the need for standardization of extraction and analysis methods. Furthermore, many studies remain limited to in vitro bioassays, with scarce investigation into the molecular mechanisms involved in antiprotozoal and antiparasitic activities. In vivo assays, toxicological studies, and pharmacokinetic tests are essential to validate the safety and efficacy of the most promising compounds. The application of modern techniques, such as molecular modeling, may accelerate the identification of therapeutic targets and optimize the structure of bioactive compounds.

### 6.13. Reproductive and Hormonal Effects

It has been observed that species of the *Cnidoscolus* genus exert effects on reproductive function, suggesting potential therapeutic or even contraceptive applications. Somade et al. [37] evaluated the effects of *C. aconitifolius* on sperm morphology and quality in rats, observing significant improvements in sperm motility and viability. Groups treated with doses of 200 and 400 mg/kg showed increases in total motility, individual motility, and sperm count. These effects were attributed to the antioxidant action of the extract, capable of protecting germ cells against oxidative stress induced by dimethylnitrosamine (DMN), thus promoting the structural and functional integrity of spermatozoa.

In a hormonal context, Iyke et al. [93] investigated the impact of the hydromethanolic extract from *C. aconitifolius* leaves in Wistar rats with streptozotocin-induced diabetes. Administration of the extract at doses of 100, 150, and 200 mg/kg resulted in a progressive and statistically significant reduction (*p* < 0.05) in luteinizing hormone (LH) levels (1.83 ± 0.23, 1.58 ± 0.26, and 0.40 ± 0.27, respectively), follicle-stimulating hormone (FSH) levels (1.80 ± 0.05, 1.50 ± 0.01, and 0.39 ± 0.01), and estradiol levels (2.28 ± 0.13, 8.03 ± 0.25, and 7.80 ± 0.18). Hormone levels decreased in a dose-dependent manner. These results suggest that the phytochemical compounds present in the extract may negatively modulate the hypothalamic–pituitary–gonadal axis, raising the hypothesis of a possible contraceptive effect of the extract, with potential application as an antifertility agent.

Complementing these findings, Tinco-Jayo et al. [67] reported the effects of spray-dried extracts from the leaves and stems of *C. diacanthus* on hormone concentrations in Holtzman rats. In females treated with 25 mg/kg of the leaf extract, a significant increase in testosterone levels was observed, while in males, doses of 100 mg/kg of the leaf and stem extracts also resulted in hormonal elevations. However, the divergent effects between sexes indicate that the hormonal action of the extracts may be gender-dependent, warranting further investigation into the specificity and safety of this effect.

Despite the results indicating potential of *Cnidoscolus* species in fertility and sex hormone modulation, knowledge gaps remain. Most studies are limited to animal models, and clinical trials are needed to confirm efficacy and safety in humans. Additionally, the mechanisms are not yet fully elucidated, particularly regarding the hormonal signaling pathways involved and the direct action of bioactive compounds on hormone receptors. Moreover, in vitro assays with Sertoli, Leydig, and granulosa cells, as well as transcriptomic and proteomic analyses, may provide more precise information about the molecular targets involved.

### 6.14. Hypolipidemic and Metabolic Activity

Guevara-Cruz et al. [44] demonstrated that the daily consumption of 500 mL of *C. chayamansa* extract for six weeks significantly reduced serum triglyceride levels (*p* < 0.05). This effect suggests that the species has potential as an adjuvant in the treatment of dyslipidemias, reinforcing its relevance in traditional medicine.

### 6.15. Hematological Parameters and Antithrombotic Activity

Researchers observed that the extract of *C. aconitifolius* (400 mg/kg) promoted significant increases in red blood cell count, hematocrit, hemoglobin, and platelet levels, suggesting stimulation of erythropoiesis and thrombopoiesis without impacting leukocyte counts [165]. In parallel, Quintal-Martínez et al. [97] reported that different leaf extracts of *C. aconitifolius* exhibited antithrombotic action. The ethanolic extract efficiently inhibited platelet aggregation, the acetonic extract significantly altered coagulation times (PT and aPTT), and the ethyl acetate extract was the most effective in clot lysis, possibly due to the presence of triterpenes and fatty acids, according to the authors. However, various mechanisms are involved in blood coagulation, and further studies are needed to elucidate the specific mechanisms of the extract.

### 6.16. Clastogenic and Antimutagenic Activities

Somade et al. [96] demonstrated the clastoprotective activity of the ethanolic extract of *C. aconitifolius*, which reduced the frequency of polychromatic micronuclei in bone marrow cells of rats exposed to DMN, attributed to its antioxidant capacity. Additionally, Loarca-Piña et al. [85] demonstrated the antimutagenic potential of the methanolic extract of *C. chayamansa*, which inhibited between 24% and 39% of chemically induced mutagenicity in *Salmonella* strains, without exhibiting toxicity. This suggests that *C. chayamansa* leaves may offer protection against genetic damage.

### 6.17. Hypotensive Activity

Manzanilla Valdez et al. [151] reported that the aqueous extract of *C. aconitifolius* leaves significantly reduced systolic (15.3%) and diastolic (23.4%) blood pressure in rats. According to the authors, the hypotensive effect of this species may be associated with the inhibition of angiotensin-converting enzyme (ACE), promoting vasodilation and consequent blood pressure reduction, highlighting the plant’s potential as a phytotherapeutic agent in the treatment of hypertension.

### 6.18. Immunomodulatory Activity

Studies conducted by Hidayati et al. [166] demonstrated that the ethanolic extract of *C. aconitifolius* leaves significantly (*p* < 0.05) increased the expression of CD4+ and CD8+ T cells in mice, especially at a dose of 400 mg/kg. This effect suggests an immunomodulatory action, possibly mediated by the presence of flavonoids with stimulating properties on the immune system.

### 6.19. Anti-Cataractogenic Activity

Bulama et al. [134] evaluated the effects of the methanolic extract of *C. aconitifolius* and its fractions in an in vitro cataract model using goat lenses. Extracts at concentrations of 250 μg/mL significantly (*p* < 0.05) increased catalase activity and total protein levels, while they reduced malondialdehyde (MDA) levels, a marker of lipid peroxidation. These effects indicate protection against lens opacification induced by oxidative stress, suggesting the potential use of the plant in the prevention or treatment of cataracts.

### 6.20. Gastroprotective Activity

The study by Olivia et al. [167] demonstrated that the methanolic extract of *C. aconitifolius* exerted a gastroprotective effect in a diclofenac-induced gastric ulcer model. The extract dose-dependently (100, 200, and 400 mg/kg body weight) reduced the mean ulcer index, gastric secretion, and acidity, while promoting mucosal regeneration. The authors attributed this effect to the phytochemical constituents present in the extract, although the mechanisms of action have not been elucidated.

### 6.21. Nephroprotective Activity

Somade et al. [37] also demonstrated that extracts from *C. aconitifolius* leaves reduced MDA levels and restored endogenous antioxidant levels (GSH, CAT, SOD, and GST) in rats with DMN-induced nephrotoxicity. The nephroprotective effect appears to be associated with the presence of phenolic compounds and flavonoids, which act by neutralizing free radicals and protecting against oxidative renal damage. The results reinforce the therapeutic potential of this plant in mitigating kidney injuries caused by toxic agents and highlight the importance of further research into its medicinal properties.

## 7. Toxicity

Despite the widespread traditional use and the scientifically evaluated therapeutic potential, toxicological information available on species of the genus *Cnidoscolus* remains limited. Some studies indicate that the consumption of certain parts of these plants may be safe when subjected to appropriate preparation methods; however, there are reports of acute toxicity associated with specific species.

In *C. aconitifolius*, although tests in animal models (rats) revealed low oral toxicity at doses up to 5.000 mg/kg, with no adverse effects such as lethality or behavioral alterations [168], studies using *Artemia salina* revealed significant toxicity of the leaf extract (LC_50_ = 74.34 µg/mL) [75]. This may reflect differences in sensitivity between experimental models and underscores the importance of employing multiple approaches for safety assessment.

Toxicity cases have also been reported in humans, as demonstrated by a case study involving accidents with *C. texanus* in Texas (USA). The hands and legs were the most affected body regions (52% and 21% of reported cases, respectively), with symptoms including skin irritation, erythema, pruritus, urticaria, edema, and lesions [169], indicating a dermatotoxic risk associated with direct contact with the plant’s spiny structures.

Regarding *C. quercifolius*, studies with leaf extracts showed no signs of acute toxicity at doses up to 2.000 mg/kg in rodents, with no observed motor or sensory alterations [109]. However, another study that administered extracts orally to albino rats (2 g/kg) reported significant biochemical alterations, despite the absence of overt toxic effects [170]. These findings suggest possible subclinical or chronic effects, highlighting the need for additional long-term investigations. Conversely, the seed oil of this species did not demonstrate significant toxicity at doses up to 5.000 mg/kg [19]. Additionally, in a study using the zebrafish (*Danio rerio*) model, it was found that the ethyl acetate and methanolic extracts of the bark exhibited an LD_50_ greater than 400 mg/kg after 96 h of exposure, and they were considered non-toxic based on the parameters evaluated [138].

For *C. chayamansa*, studies indicate a favorable safety profile in animal models, with an LD_50_ above 5 g/kg in acute toxicity tests [96]. Additional evaluations of acute and subacute toxicity revealed that the extracts administered via the intragastric route presented an LD_50_ greater than 2 g/kg. Furthermore, after 28 days of continuous administration at a dose of 1 g/kg, no relevant adverse effects were observed, such as weight gain or significant changes in important biochemical parameters, including kidney and liver damage [9].

Despite these initial findings, few studies have comprehensively evaluated the toxic effects of species within the genus *Cnidoscolus*. Given the frequent use of these plants in both food and traditional medicine, their safety profile still warrants specific attention, particularly considering the presence of potentially toxic compounds such as cyanogenic glycosides identified in some species. Therefore, there is an urgent need for more robust toxicological studies, including assessments of chronic, subchronic, and genotoxic toxicity, as well as evaluations of pharmacokinetic and pharmacodynamic parameters. These efforts are essential to establish safe consumption limits and to identify potential risks associated with different plant parts, routes of administration, and preparation methods.

## 8. Conclusions

Various species in the genus *Cnidoscolus* have been considered medicinal and nutritious in recent years, rich in compounds that promote health and well-being. For these reasons, *Cnidoscolus* species have attracted the attention of scientists throughout the years, with dedication to deepening the chemical and biological properties.

It has been noticed that a wide range of therapeutic potentials has been demonstrated by extracts and isolated compounds from species of the genus *Cnidoscolus*, corroborating their empirical use and highlighting their potential in drug development. However, despite the promising findings, most of the available results are based on in vitro or animal models, with a scarcity of clinical trials validating their efficacy and safety in humans, as well as investigations detailing the molecular mechanisms related to the observed activities.

Based on the studies analyzed, it was evident that there is a lack of standardization of extracts and insufficient identification of the bioactive compounds responsible for the observed activities. Moreover, many investigations focus predominantly on a few species, such as *Cnidoscolus aconitifolius* and *C. chayamansa*, while others, including *C. infestus*, *C. obtusifolius*, *C. multilobus*, *C. diacanthus*, and *C. tubulosus*, remain underexplored from chemical, pharmacological, and toxicological perspectives, despite their reported use in traditional medicine.

To advance the scientific understanding and therapeutic applications of these species, it is essential that future research includes in-depth mechanistic and pharmacodynamic studies to elucidate how these substances interact within biological systems. Additionally, to fully realize the potential of the *Cnidoscolus* genus, there is an urgent need for further investigations on these lesser-studied species, particularly regarding the properties suggested by ethnopharmacological reports.

Equally important is the conduction of short- and long-term toxicological studies to ensure the safety of continued use, thereby contributing to future clinical validation and functional applications in human nutrition. Such advancements are crucial for establishing a rational and evidence-based use of *Cnidoscolus* spp. in integrative medicine, ultimately enhancing the therapeutic and nutritional potential of these native and traditionally used plants.

## Figures and Tables

**Table 2:** Phenolic compounds identified and isolated from *Cnidoscolus* species (2000 to 2025).

Species Name	Isolated and/or Identified Chemical Compounds	Plant Part	Method of Identification/Isolation	Reference
*Cnidoscolus chayamansa* McVaugh (Chaya)	Rosmarinic acid	Leaves	HPLC-MS/MS QQQ	[81]
	Epigallocatechin gallate			
	Rutin		HPLC-MS/MS QQQ HPLC-DAD	[81,97]
	Naringenin		HPLC-MS/MS QQQ	[81]
	Chlorogenic acid		HPLC-MS/MS QQQ HPLC-DAD	[81,97]
	Ferulic acid			
	Protocatechuic acid			
	Astragalin		HPLC-MS/MS QQQ	[81]
	Caffeic acid		HPLC, DAD, MS/MS QQQ	[81,97]
	Myristic acid		HPLC-MS/MS QQQ	[81]
	Riboflavin			
	*β*-carotene			
	Quercetin		HPLC, DAD, MS/MS QQQ	[81,97]
	Palmitic acid			
	4-hydroxybenzoic acid	Leaves	HPLC-DAD	[84]
	*p*-coumaric acid			
	Sinapic acid			
	Ellagic acid			
	Catechin			
	Hesperidin			
	Gallocatechin gallate			
	Naringenin			
	Vanillin			
	3-*O*-rhamnosyl glycoside			
	3-*O*-galactoside			
	3-*O*-glucoside			
	3-*O*-rhamnoside			
	3-*O*-rhamnosyl galactoside			
	7-*O*-glucoside,			
	3-*O*-rhamnosyl galactoside			
	7-*O*-rhamnoside			
	Quercetin glycosides			
	3-*O*-rhamnosyl glucoside			
	*β*-sitosterol	Leaves	NMR, GC-MS, CC-NP, TLC	[104,105]
	Kaempferol-3,7-dimethyl ether	Leaves	NMR (^1^H, ^13^C), GC-MS, TLC, CC	[9]
	5-hydroxy-7,3′4′-trimethoxyflavanone			
	Quercetin	Leaves	NMR (^1^H, ^13^C), GC-MS, TLC, CC	[9,11]
	Kaempferol stigmastadiene			
*Cnidoscolus aconitifolius* (Mill.) I.M. Johnst. (Chaya)	3,4-dihydroxybenzoic acid	Leaves	HPLC	[44]
	Chlorogenic acid			
	Ellagic acid	Leaves	LC-ESI-MS/MS	[42]
	Ferulic acid		LC-ESI-MS/M, UPLC-DAD-QToF/MS-ESI	[42,82]
	Gallic acid		LC-ESI-MS/MS	[42]
	Rosmarinic acid		LC-ESI-MS/M, UPLC-DAD-QToF/MS-ESI	[42,76]
	Salicylic acid			
	Sinapic acid			
	Syringic acid			
	Vanillic acid			
	Apigenin			
	Catechin			
	Chrysin			
	Epicatechin			
	Eriodictyol			
	Fustin			
	Galangin			
	Hispidulin			
	Isorientin			
	Myricetin			
	Naringenin			
	Pinocembrin			
	Taxifolin			
	Vitexin			
	Sinapaldehyde			
	Syringaldehyde			
	Vanillin			
	Scopoletin			
	Umbelliferone			
	Carnosol			
	Hydroxybenzoic acid	Leaves	UPLC-DAD-QToF/MS-ESI	[76]
	Hydroxyphenylacetic acid			
	Rutin		LC-ESI-MS/MS, UPLC-DAD-QToF/MS-ESI	[42,76]
	Epicatechin		UPLC-DAD-QToF/MS-ESI	[76]
	Epigallocatechin gallate			
	Resveratrol			
	Quercetin			
	Caffeic acid			
	*p*-coumaric acid			
	Hispidulin sulphate	Leaves	HPLC/MS	[98]
	Eucalyptin			
	Polyanxanthone C			
	Cadensin G			
	Parvixanthone D			
	(epi)Gallocatechin di-*O*-gallate			
	(epi)Catechin di-*O*-gallate			
	Fraxetin			
	Acutifolin D			
	Hamaudol			
	Phenylmalonic acid	Leaves	CC, GC-MS	[99]
	Amentoflavone	Leaves	HPLC-DAD	[10]
	Hesperidin			
	Protocatechuic acid			
	Dihydromyricetin			
	Quercitrin			
*Cnidoscolus quercifolius* Pohl (Favela)	Thymol	Leaves	GC-MS	[110]
	*O*-cresol methyl ether	Flowers		
	Syringic acid	Seeds	UHPLC	[19]
	Ellagic acid			
	Quercetin			
	Eugenol			
	Vanillin			
	Vanillic acid			[19,113]
	Gallic acid	Seeds	UHPLC	[113]
	Catechin			
*Cnidosculos texanus* (Müll.Arg.) Small	Aromadendrin 7-*O*-(3″,6″-di-*O*-p-E-coumaroyl-*β*-glucopyranoside)	Leaves and fruits	LC/MS/MS	[118]
	Naringenin 7-*O*-(4″-*O*-p-Z-coumaroyl-*β*-glucopyranoside)			
	Aromadendrin7-*O*-(4″-*O*-p-E-coumaroyl-*β*-glucopyranoside)			
	Naringenin 7-*O*-(4″-*O*-p-E-coumaroyl-*β*-glucopyranoside)			
	Naringenin 7-*O*-(3′-*O*-p-E-coumaroyl-*β*-glucopyranoside)			
	Naringenin 7-*O*-(3″-*O*-p-E-coumaroyl-*β*-glucopyranoside)			
	Naringenin 7-*O*-(3″,6″-di-*O*-p-E-coumaroyl-*β*-glucopyranoside)			
	Apigenin 7-*O*-(3″,6″-di-*O*-p-E-coumaroyl-*β*-glucopyranoside)			
	Apigenim 7-*O*-(6″-*O*-p-E-coumaroyl-*β*-glucopyranoside)			
	Apigenim 7-O (4″,6″-di-*O*-p-E-coumaroyl-*β*-glucopyranoside)			
	Manghaslin			
	Quercetin 3-neohesperidoside			
	Kaempferol 3-o-α-rhamnosyl-(1-2)-O-[α —rhamnosyl-(1-6)]-*β*-glucopyranoside			
	Kaempferol 3-neohesperidoside			
	Rutin			
	6-hidroxy-7-methoxycoumarin			
	6-methoxy-7-hidroxycoumarin			
	5,7-dimethoxy-6-hidroxy-coumarin			
	3-(4-ethoxyphenyl)-2-propenoic acid			
	p-coumaric acid			
	Ferulic acid			

Legend: Nuclear Magnetic Resonance (NMR), Column Chromatography (CC), Mass Spectrometry (MS), Diode Array Detector (DAD), Gas Chromatography–Mass Spectrometry (GC-MS), High-Performance Liquid Chromatography (HPLC), Liquid Chromatography–Tandem Mass Spectrometry (LC-MS/MS), Normal-Phase Column Chromatography (CC-NP), Centrifugal Partition Chromatography (CPC), Ultra-High-Performance Liquid Chromatography–Diode Array Detection–High-Resolution Tandem Mass Spectrometry (UHPLC–DAD–HRMS/MS), High-Performance Liquid Chromatography–Triple Quadrupole Tandem Mass Spectrometry (HPLC–MS/MS QQQ).

## Data Availability

No new data were created or analyzed in this study. Data sharing is not applicable to this article.

## Supplementary Materials

The following supporting information can be downloaded at: https://www.mdpi.com/article/10.3390/foods14122092/s1, Table S1. Chemical compounds identified and isolated from species of the genus *Cnidoscolus* and their investigated activities (2000 to 2025).
