# Supplementary material for: Nutritional Value, Ethnopharmacology, Chemistry, and Biological Activities of Species of the Genus Cnidoscolus: An Updated Review
Source: Foods. 2025 Jun 13;14(12):2092. doi: 10.3390/foods14122092 (PMC12192014; doi:10.3390/foods14122092)
Supplement: Supplementary file 1 [file foods-14-02092-s001.zip › foods-3644085-supplementary.pdf]

Table S1. Chemical compounds identified and isolated from species of the genus *Cnidoscolus* and their investigated activities (2000 to 2025).

Species name	Isolated and/or identified chemical compounds	Plant part	Method of identification/isolation	Biological activities presented by the compounds	Reference
Cnidoscolus chayamansa McVaugh (Chaya)	Rosmarinic acid	Leaves	HPLC-MS/MS QQQ		[81]
	Epigallocatechin gallate				
	Rutin		HPLC-MS/MS QQQ HPLC-DAD		[81,84]
	Naringenin				[81]
	Chlorogenic acid		HPLC-MS/MS QQQ HPLC-DAD		[81,84]
	Ferulic acid				
	Protocatechuic acid				
	Astragalin		HPLC-MS/MS QQQ		[81]
	Caffeic acid				
	Myristic acid				
	Riboflavin				
	β-carotene				
	Quercetin				
	Palmitic acid				
	4-hydroxybenzoic acid	Leaves	HPLC-DAD		[84]
	Caffeic acid				
	p-coumaric acid				
	Sinapic acid				
	Ellagic acid				
	Catechin				
	Hesperidin				
	Gallocatechin gallate				

	Quercetin				
	Naringenin				
	Vanillin				
	Choline				
	Trigonelline				
	Nicotinic acid				
	Palmatine				
	Sitsirikin				
	Dihydrositsirikine				
	Vinblastine				
	Vindoline				
	Catharanthine				
	Vinleurosine				
	Phytolaccagenic acid				
	28-*O*-*β*-D-glucopyranosyl ester				
	Kaempferol glycosides	Leaves	HPLC, GC		[83,88]
	3-*O*-rhamnosyl glycoside				
	3-*O*-galactoside				
	3-*O*-glucoside				
	3-*O*-rhamnoside				
	3-*O*-rhamnosyl galactoside				
	7-*O*-glucoside,				
	3-*O*-rhamnosyl galactoside				
	7-*O*-rhamnoside				
	Quercetin glycosides				
	3-*O*-rhamnosyl glucoside,				
	Phytol	Leaves	GC-MS		[104]

	Squalene				
	γ-Tocopherol				
	δ-Tocopherol				
	β-amyrin				
	Lupeol acetate		NMR, GC-MS, CC-NP, TLC		[104,105]
	β-Sitosterol		CG-MS		[104]
	Lupeol		NMR, GC-MS, CC-NP, TLC		[105]
	Octacosane				
	Andstigmast-4-en-3-one				
	β-amyrenone				
	β-amyrin acetate				
	Moretenone				
	Ergost-5-en-3-ol				
	Stigmasterol				
	β-sitosterol				
	Moretenol	Leaves	NMR (^{1}H, ^{13}C), GC-MS, TLC, CC	Anti-inflammatory and antiprotozoal activity	[9]
	Moretenyl acetate			Antiprotozoal activity	
	Kaempferol-3,7-dimethyl ether			Anti-inflammatory and antiprotozoal activity	
	5-hydroxy-7,3′4′-trimethoxyflavanone			Antiprotozoal activity	
	Quercetin	Leaves	NMR (^{1}H, ^{13}C), GC-MS, TLC, CC		[9,11]
	Kaempferol stigmastadiene,				
	β-amyrin acetate,				
	Amyrenone				
	β-sitosterol				

	β-D-glucopyranoside(1R)-O-isopropyl 6-*O*-(2,3,4-tri-O-acetyl-*β*-D-xylopyranosyl)-2,3,4-triacetate		CC, TLC, CG-MS, HPLC, NMR (^{1}H)		[11]
Cnidoscolus aconitifolius (Mill.) I.M. Johnst. (Chaya)	Quercetin	Leaves	HPLC		[44]
	Kaempferol				
	3.4-dihydroxybenzoic acid	Leaves	LC-ESI-MS/MS		[42]
	4-aminobenzoic acid				
	4-hydroxymethyl benzoic acid				
	Cinnamic acid				
	Chlorogenic acid		LC-ESI-MS/M, UPLC-DAD-QToF/MS-ESI		[42, 76]
	Ellagic acid		LC-ESI-MS/MS		[42]
	Ferulic acid		LC-ESI-MS/M, UPLC-DAD-QToF/MS-ESI		[42, 76]
	Gallic acid				
	Mandelic acid		LC-ESI-MS/MS		[42]
	p-anisic acid				
	Rosmarinic acid				
	Salicylic acid				
	Sinapic acid				
	Syringic acid				
	Vanillic acid				
	Apigenin				
	Catechin				
	Chrysin				
	Epicatechin				
	Eriodictyol				
	Fustin				
	Galagngina				

	Hispidulin				
	Isorientin				
	Myricetin				
	Naringenin				
	Pinocembrin				
	Rutin				
	Taxifolin				
	Vitexin				
	Sinapaldehyde				
	Syringaldehyde				
	Vanillin				
	Scopoletin				
	Umbelliferone				
	Carnosol				
	Hydroxybenzoic acid	Leaves	UPLC-DAD-QToF/MS-ESI		[76]
	Hydroxyphenylacetic acid				
	Rutin				
	Epicatechin				
	Epigallocatechin gallate				
	Resveratrol				
	Quercetin				
	Sinapic acid				
	Caffeic acid				
	p-coumaric acid				
	Tieguanyin	Leaves	HPLC/MS		[98]
	Hispidulin sulphate				
	Eucalyptin				

	Polyanxanthone C				
	Cadensin G				
	Parvixanthone D				
	(epi) Gallocatechin di-*O*-gallate				
	(epi) Catechin di-*O*-gallate				
	Fraxetin				
	Acutifolin D				
	Hamaudol				
	Moreollic acid				
	Phenylmalonic acid	Leaves	CC, GC-MS		[99]
	Benzene acetic acid				
	3-Oxo-4-phenylbutyronitrile				
	Spiro-(2,4)-hepta-4, 6-diene				
	4 nitrosophenyl-β-phenyl propionate				
	Benzene propanoic acid				
	3-phenyl-propionic acid				
	Tetradecanoic acid				
	Octadecanoic acid				
	n-Hexadecanoic acid				
	Eicosanoic acid				
	Pentadecanoic acid				
	Cyclo-tetradecane				
	5-octadecene				
	7-hexadecene				
	5-eicosene				
	Do-decanoicacid-1,2				
	3-propanetriyl ester				

	Do-decanoicacid				
	1-hydroxy methyl 1				
	2-diyl ester				
	Do-decanoicacid				
	Ethenyl ester				
	2(4H)-Benzofuranone	Leaves	GC-MS		[100]
	5,6,7,7a-tetrahydro-4,4				
	7a-trimethyl-				
	(R)- Dodecanoic acid				
	3-Eicosene				
	(E)- 4-Isobenzofuranol				
	Octahydro-3a				
	7a-dimethyl				
	6-Methyl-cyclodec-5-enol				
	Tetradecanoic acid				
	3-Chloropropionic acid				
	Heptadecyl ester				
	2-Pentadecanone				
	6,10,14-trimethyl				
	Methyl ester				
	Cyclopropanenonanoic acid				
	2-[(2-butylcyclopropyl) methyl]-				
	Phytol, 9,12,15-Octadecatrienoic acid				
	Ethyl 14-methyl-hexadecanoate				
	n-Hexadecanoic acid				
	9,12-Octadecadienoic acid				
	9,12,15-Octadecatrienoic acid				

	(Z,Z,Z)-9,12,15-Octadecatrienoic acid				
	(Z,Z,Z)- Octadecanoic acid				
	Hexadecanoic acid				
	1,1-dimethylethyl ester				
	3,7,11,15-Tetramethyl-2-hexadecen-1-ol				
	Butyl 9,12,15-octadecatrienoate				
	9-Octadecenamide				
	(Z)-Butyl 9,12,15-octadecatrienoate				
	Butyl 9,12,15-octadecatrienoate				
	9,12,15-Octadecatrienoic acid				
	2,3-dihydroxypropyl ester				
	(Z,Z,Z)-Hexadecanoic acid				
	2-hydroxy-1-(hydroxymethyl)ethyl ester				
	Tetrahydrosmilagenin				
	Squalene				
	15-chloro				
	(Z,Z,Z)-methyl ester				
	9,12,15-Octadecatrienoic acid				
	2,3-dihydroxypropyl ester				
	(Z,Z,Z)-cis-5,8,11-Eicosatrienoic acid				
	Trimethylsilyl ester				
	I-Propyl 9,12,15-octadecatrienoate				
	2-Myristynoyl-glycinamide				
	2H-3,9a-Methano-1-benzoxepin				
	2-Myristynoyl-glycinamide				
	2H-3,9a-Methano-1-benzoxepin				
	Cholestan-6-one				

	3-methoxy-(3.beta.,5.alpha)				
	Pseudosmilagenin				
	2H-3,9a-Methano-1-benzoxepin				
	α-Tocopheryl acetate				
	Amentoflavone	Leaves	HPLC-DAD		[10]
	Hesperidin				
	Protocatechuic acid				
	Dihydromyricetin				
	Quercitrin				
	Xylose	Leaves	-		[100]
	Arabinose				
	Mannose				
	Galactose				
	Glucose				
	Uronic acid				
	Palmitic acid	Leaves	GC-MS		[97]
	Ethyl palmitate				
	Phytol				
	α-linolenic acid				
	Squalene				
	Vitamin E				
	Stigmasterol				
	β-Amyrin				
	β-Amyrin acetate				
	Lupeol acetate				
	Amyrenone	Leaves	TLC, GC-MS		[106]
	γ-amyrin acetate				

	Icosahydropicen-3-ol	Leaves	NMR (^{1}H, ^{13}C), COSY, HMBC, HSQC e LC-MS		[103]
	5*β*-pregnane				
Cnidoscolus quercifolius Pohl (Favela)	*α*-Terpineol	Leaves	GC-MS		[113]
	1,4-Cineole				
	Cis-Arbusculone				
	Pinene trans-hydrate				
	Dihydrolinalool				
	Menthone				
	Tetrahydrolavandulol				
	Neo-dihydrocarveol				
	Shisofuran				
	Cis-4-Caranone				
	Citronellol				
	Thymol				
	Verbenyl trans-acetate				
	Silphiperfol-4,7(14)-diene				
	(Z)-*β*-Damascone				
	α-Funebrene				
	Sesquitujene				
	(E)-*β*-Farnesene				
	Methyl-*β*-(E)-ionol				
	(E)-*β*-Ionene				
	Bicyclogermacrene				
	β-Bisabolene				
	α-Cadinene				
	(E)-Nerolidol				
	Curcumenol				

	Acid Vetivenic				
	Cryptomeridiol				
	β-Vetivone				
	11,12-Dihydroxyvalencene				
	Phytol				
	Isophytol				
	γ-terpinene	Flowers			
	α-Pinene				
	Sabinene				
	β-Pinene				
	Myrcene				
	Dehydroxy-trans-linalool oxide				
	Meta-mentha1-(7),8-diene				
	δ-2-Carene				
	α-Phellandrene				
	O-cresol methyl ether				
	Iso-Sylvestrene				
	δ-3-Carene				
	α-Terpinene				
	Limonene				
	Trans-Pinene hydrate				
	Dihydrolinalool				
	Cis-Verbenol				
	Cis-*β*-Terpineol				
	Neo-3-Tujanol				
	Cis-Dihydro-β-terpineol				
	Tetrahydrolavandulol				

	α-Terpineol				
	14-cedranediol	Bark			[113]
	Isopimarone-9 (11)				
	15-diene, beyerene				
	Isohibaene				
	Cembrene,				
	Palmitic acid				[113,121]
	13-epi-dolabradiene				[113]
	Polygodial				
	Phyllocladene				
	Manool,				
	Abietadiene				
	Linoleic acid				[19,113, 121]
	Oleic acid				[113,121]
	Sandaracopimarinal				[113]
	Phyllocladanol				
	Syringic acid	Seeds	UHPLC		[19]
	Ellagic acid				
	Quercetin				
	Eugenol				
	Vanillin				
	Vanillic acid				
	Myristic	Seeds	CG, FID, HPLC, UV–vis		[107,104,111, 112]
	Palmitic				
	Stearic				
	Oleic				
	Arachidic				

	Behenic		GC, FID, UV-Vis		[112]
	Gallic acid	Seeds	UHPLC		[116]
	Catechiz				
	Vanillic acid				
	Margaric	Seeds	-		[67]
	Cis-11-eicosenoic				
	Alpha linolenic				
	Faveline	Root bark	HPLC-DAD, NMR (^1H, ^{13}C),	Antiproliferative effect	[114]
	O-Methyl faveline				
	Deoxofaveline			Antiproliferative effect	
	Neofavelanone				
	β-ionone	Leaves and stem bark	GC-MS		[115]
	Dihydroactinidiolide				
	1-tridecene				
	Tetradecane				
	2,3,7-trimethyl-decane				
	2-methyl-tetradecane				
	3-methyl-tetradecane				
	2-methyl-hexadecan-1-ol				
	Pentadecane				
	5-methyl-pentadecane				
	2-methyl-pentadecane				
	3-methyl-pentadecane				
	3-hexadecene				
	1-hexadecene				
	Hexadecane				
	3-hexyl-1,1,2-trimethyl-cyclobutane				

	2,6,10-trimethyl-pentadecane				
	1-cyclohexyl-decane				
	1-decyl-cyclopentane 2-methyl-hexadecane				
	2-methyl-heptadecane				
	2-phenyl-dodecane				
	Heptadecane				
	2,6,10,14-tetramethyl-pentadecane				
	7-methyl-hexadecane				
	4-ethyl-heptadecane				
	3-methyl-heptadecane				
	1-octadecene				
	Octadecane				
	2,6,10,14-tetramethyl-hexadecane				
	4-cyclohexyl-tridecane				
	Hexadecan-1-ol				
	Nonadecane				
	Octasane				
	3-methyl-nonadecane				
	2-methyl-eicosane				
	1-tricosene				
	1-nonadecene				
	Eicosane				
	Octadecan-1-ol				
	Heneicosan-1-ol				
	Heneicosane				
	7-hexyl-eicosane				
	Docosane				

	7-hexyl-docosane				
	Pentacosane				
	Nonacosane				
	Hexacosane				
	Heptacosan-1-ol				
	Tetracontane				
	Tetratetracontane				
	Sandaracopimaradiene				
	13-methyl-17-norkaur-15-ene				
	Kaur-16-ene				
	Dehydroabietane				
	Lupeol				
	Diploptene				
	Favelol	Stem bark	CPC, HPLC, UHPLC–DAD–HRMS/MS		[116]
	Neofavelanone				
	Isofavelol				
	Faveline				
	Favelanone				
	Phyllacanthone B				
	Phyllacanthone			Antiproliferative activity	
	Deoxofaveline				
	Linamarin	Leaves	IR, MS e RMN	Antimicrobial, antiprotozoal and antiparasitic activity	[122]
	Trans-cinnamic acid				
	Steroids				
	Triterpenes				
	Lupeol-3*β*-*O*-cinnamate		GC-MS	Antibacterial activity	[123]

	Lupeol-3*β* *O*-dihydrocinnamate	Leaves and Stem barks		Antibacterial activity	
	Phylcanthone	Stem barks	GC-MS, TLC, NMR (^1H, ^{13}C), CC	Antibacterial activity	[113,124,125]
	Lupeol-3β-O-cinnamate		NMR (^1H, ^{13}C), CC, TLC		[125]
	Lupeol-3*β*-*O*-dihydrocinnamate				
	Lupeol-3*β*-*O*-hexanoate				
	Favelanone				
Cnidoscolus phyllacanthus (Mull.Arg.) Pax & K.Hoffm (synonym of *C. quercifolius*) (Favela)	Stearic acid	Seeds	-		[121]
Cnidoscolus urens (L.) Arthur (Cansação)	Palmitic acid	Seeds	GC-MS		[119]
	Stearic acid				
	Oleic acid				
	Linoleic acid				
	n-Hexadecanoic acid	Leaves and stems	GC-MS		[120]
	Nonadecanoic acid				
	Phytol				
	9,12-Octadecadienoic acid				
	Ethyl ester				
	Squalene				
Cnidoscolus tehuacanensis Brekon LC.	*β*-amyrin acetate	Leaves	GC-MS, CC-NP, HPLC		[117]
	Germanicol				
	Lupeol acetate				

	β-amyrenone				
	Betulin				
	Lanosterol acetate				
	β-amyrenone				
	β-amyrin				
	Lupenone				
	1-octacosanol				
	β-sitosterol				
	Lupeol				
	Stigmast 4-en-3-one				
Cnidosculos texanus (Müll.Arg.) Small	Aromadendrin 7-*O*-(3″,6″-di-*O*-p-E-coumaroyl-*β*-glucopyranoside)	Leaves and fruits	LC/MS/MS		[145]
	Naringenin 7-*O*-(4″-*O*-p-Z-coumaroyl-β-glucopyranoside)				
	Aromadendrin7-*O*-(4″-*O*-p-E-coumaroyl-*β*-glucopyranoside)				
	Naringenin 7-*O*-(4″-*O*-p-E-coumaroyl-*β*-glucopyranoside)				
	Naringenin 7-*O*-(3′-*O*-p-E-coumaroyl-*β*-glucopyranoside)				
	Naringenin 7-*O*-(3″-*O*-p-E-coumaroyl-*β*-glucopyranoside)				
	Naringenin 7-*O*-(3″,6″-di-*O*-p-E-coumaroyl-*β*-glucopyranoside)				
	Apigenin 7-*O*-(3″,6″-di-*O*-p-E-coumaroyl-*β*-glucopyranoside)				
	Apigenim 7-*O*-(6″-*O*-p-E-coumaroyl-*β*-glucopyranoside)				

	Apigenim 7-O (4″,6″-di-*O*-p-E-coumaroyl-β-glucopyranoside)				
	Manghaslin				
	Quercetin 3-neohesperidoside				
	Kaempferol 3-o-*α* -rhamnosyl-(1-2)-O-[*α* — rhamnosyl-(1-6)]- β -glucopyranoside				
	Kaempferol 3-neohesperidoside				
	Rutin				
	6-hidroxy-7-methoxycoumarin				
	6- methoxy -7- hidroxycoumarin				
	5,7-dimethoxy-6-hidroxy-coumarin				
	3-(4-ethoxyphenyl)-2propenoic acid				
	p-coumaric acid				
	Ferulic acid				
	Lupeol 3-acetate				
	Lupeol				
	Germanicol 3-acetate				
	Germanicol				
	Daucosterol				
Cnidosculos spinosus Lundell	3-oxo-hop-22(29)-ene	Leaves	UV, IR, NMR, CC, TLC	Anti-inflammatory and antiparasitic activity	[126]
	3β-hydroxy-hop-22(29)-ene				
	3β-acetoxy-hop-22(29)-ene				
	3-oxo hop-22(29)-ene				
Cnidoscolus souzae McVaug (Faveleira)	7-deoxynimbidiol	Roots	NMR (^{1}H, ^{13}C), COSY, HSQC, HMBC, HPLC, CC, VLC	Anti-nociceptive activity	[127]

Legend: Nuclear Magnetic Resonance (NMR), Column Chromatography (CC), Mass Spectrometry (MS), Diode Array Detector (DAD), Gas Chromatography–Mass Spectrometry (GC-MS), Correlation Spectroscopy (COSY), High-Performance Liquid Chromatography (HPLC), Vacuum Liquid Chromatography (VLC), Heteronuclear Single Quantum Coherence Spectroscopy (HSQC), Heteronuclear Multiple Bond Correlation Spectroscopy (HMBC), Infrared Spectroscopy (IR), Ultraviolet–Visible Spectroscopy (UV–Vis), Thin-Layer Chromatography (TLC), Liquid Chromatography–Tandem Mass Spectrometry (LC-MS/MS), Normal-Phase Column Chromatography (CC-NP), Centrifugal Partition Chromatography (CPC), Ultra-High-Performance Liquid Chromatography–Diode Array Detection–High-Resolution Tandem Mass Spectrometry (UHPLC–DAD–HRMS/MS), Ultra-Performance Liquid Chromatography–Diode Array Detection–Quadrupole Time-of-Flight Mass Spectrometry with Electrospray Ionization (UPLC–DAD–QToF/MS–ESI), Liquid Chromatography–Electrospray Ionization–Tandem Mass Spectrometry (LC–ESI–MS/MS), High-Performance Liquid Chromatography–Triple Quadrupole Tandem Mass Spectrometry (HPLC–MS/MS QQQ).
